# Lift Me Up by Looking Down: Social Comparison Effects of Narratives

**DOI:** 10.3389/fpsyg.2018.01889

**Published:** 2018-10-18

**Authors:** Stefan Krause, Silvana Weber

**Affiliations:** Human-Computer-Media Institute, University of Würzburg, Würzburg, Germany

**Keywords:** self, self-concept, transportation, identification, experience taking, narratives, social comparison

## Abstract

Stories are a powerful means to change recipients’ views on themselves by being transported into the story world and by identifying with story characters. Previous studies showed that recipients temporarily change in line with a story and its characters (assimilation). Conversely, assimilation might be less likely when recipients are less identified with story protagonists or less transported into a story by comparing themselves with a story character. This may lead to changes, which are opposite to a story and its characters (contrast). In two experiments, we manipulated transportation and experience taking via two written reviews (Experiment 1; *N* = 164) and by varying the perspective of the story’s narrator (Experiment 2; *N* = 79) of a short story about a negligent student. Recipients’ self-ratings in comparison to others, motives, and problem-solving behavior served as dependent variables. However, neither the review nor the perspective manipulation affected transportation or experience taking while reading the story. Against our expectations, highly transported recipients (in Study 1) and recipients with high experience taking (in Study 2) showed more persistency working on an anagram-solving task, even when controlling for trait conscientiousness. Our findings are critically discussed in light of previous research.

## Introduction

In daily life, people are exposed to a great number of narratives, for example, in advertising, books, or movies. Narratives let us experience the personal history of people with various backgrounds that are different from our own. This can broaden our understanding of other people’s struggles and achievements, who we would have never met (Sestir and Green, 2010) – or they can feature people who we might rather look down at (Mares and Cantor, 1992). Thereby, narratives are potentially powerful means to produce temporal changes in recipients’ selves by giving them the experience of different lives and personas. The influence of stories is often attributed to their power to transport us to other places (*transportation*; Gerrig, 1993; Green and Brock, 2000). Furthermore, recipients identify with story characters (Oatley, 1994; Cohen, 2001) by temporarily simulating their thoughts, emotions, and goals (*experience taking;* Kaufman and Libby, 2012). Due to these processes, recipients’ selves can temporarily change in line with either the theme of the narrative or with specific traits of story characters, a process called *assimilation* (Appel, 2011; Richter et al., 2014). However, stories do not always work like a simple “hypodermic needle” that injects a different self into its recipients. Instead, a story and its protagonists might also serve as a standard of social comparison (Festinger, 1954; Biernat, 2005). As the result of a social comparison process (particularly with a lower comparison standard), recipients’ self-concepts, motives, and even their behaviors might temporarily change by contrasting themselves away from traits and behavior depicted in a story. These *contrast effects* are expected when recipients have a mindset that leads them to compare themselves with a story protagonist (Mussweiler, 2007; Appel, 2011), and when they compare themselves downward with others who are worse off, in order to feel better about themselves (Mares and Cantor, 1992). Up until now, downward social comparison with protagonists and potential (contrast) effects on recipients’ selves, as well as the mediating role of transportation and experience taking in the process are not well understood. Acknowledging this research gap, we took an experimental approach to manipulate transportation (Study 1) and experience taking (Study 2). The goal of the present research was to examine potential outcomes of contrast effects and downward social comparison with an incompetent protagonist (Study 1) and a negligent protagonist (Study 2).

## Theory

### Effects of Narratives on Recipients

To date, most research regarding narratives and how they influence the self is guided by the idea that recipients’ beliefs become similar to aspects of the story by being immersed into the story (Green and Brock, 2000) or by temporarily assuming protagonists’ characteristics (Cohen, 2001; Kaufman and Libby, 2012). Furthermore, there is some empirical evidence that stories could even temporarily shift recipients’ self-perceptions, motives, and behavior in line with the story and its characters, a process called *assimilation* (Sestir and Green, 2010; Appel, 2011; Gabriel and Young, 2011; Richter et al., 2014). According to Appel (2011), reading a highly transporting story and having a close connection to its protagonist should lead to assimilation effects. The central idea of transportation (Green and Brock, 2000) is based on a metaphorical journey into the story. During this journey, recipients may temporarily lose access to their real world surroundings, and when they return, they are changed by this intense experience (Gerrig, 1993).

Shedlosky-Shoemaker et al. (2011) and Bacherle (2015) experimentally manipulated transportation by asking participants to read a positive or negative review prior to reading the story. Through the review, people form a specific mindset and expectations about the upcoming story, which subsequently influence transportation while reading, listening to, or watching a story (Shedlosky-Shoemaker et al., 2011). This approach has also been successfully applied in the area of health narratives. In an experimental study by Gebbers et al. (2017), transportation was manipulated using negative vs. positive reviews before watching a video-clip about a car accident caused by a drunken driver. Highly transported participants (positive review condition) rated the risk severity of drunk driving significantly higher compared to less transported participants (negative review condition).

Complementary to transportation, which describes a more holistic involvement with the story, *identification* or *experience taking* particularly refer to character involvement. *Experience taking* (Kaufman and Libby, 2012) or *identification* (Cohen, 2001) describe the imaginative process of temporally simulating the perspective of a character in a story (Dal Cin et al., 2007; Sestir and Green, 2010). As both concepts, experience taking and identification, are highly similar, we decided to employ the term experience taking throughout this manuscript. Sestir and Green (2010) experimentally manipulated experience taking and transportation via written instructions before watching a movie (e.g., high experience taking: “observe the clip as if you were the main character in the clip”; p. 277) in order to show assimilation effects. Participants with high experience taking and transportation scores showed stronger trait shifts in a Me/Not-Me task in line with the story character than participants who identified less with the story character and who were less transported.

Manipulations of experience taking include the variation of the narrative voice of a story. A first-person voice entails the main character, who narrates the story from his/her point of view, whereas in a third-person voice story, an independent observer serves as a more distant narrator of the story events and the characters. Kaufman and Libby (2012) showed that a story written from a first-person voice depicting a main character of the same group as the reader (i.e., in-group) led to higher experience taking values compared to a story written in a third-person voice with an out-group protagonist.

**FIGURE 1:**
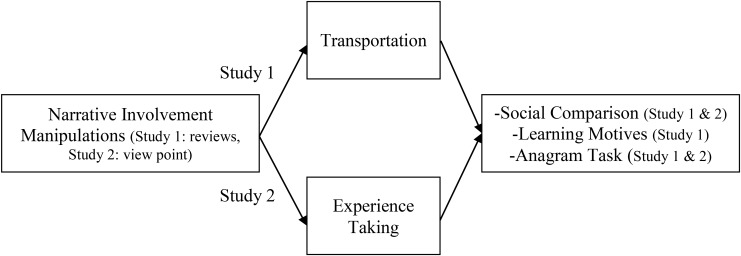
Proposed model of assimilation vs. contrast effects for both studies.

It is important to note that both processes of narrative involvement – transportation and experience taking – are considered to be largely intertwined, yet distinguishable (Moyer-Gusé, 2008; Brown, 2015). A single experimental approach that aims at the manipulation of only one of these processes might not be sufficient to describe the specific processes of narrative involvement. On this account, we used two different manipulations that aimed at varying transportation (Study 1) and experience taking (Study 2), respectively. **Figure 1** gives an overview of our complete model and assumptions.

Recipients’ engagement into a story and its characters are central mediators that might explain changes in participants’ selves in line with a story (assimilation effects). Yet, what happens if recipients have a more distant view toward a story and its protagonist? Both approaches, transportation and experience taking, do not explicitly address this open question. Under conditions of feeling less transported into a story and low experience taking with the protagonist, we expected recipients to compare themselves with others to gain relevant information about oneself (Green, 2005).

### Social Comparison Framework

Social comparison theory (Festinger, 1954) posits that people strive to gain self-knowledge by comparing themselves with similar others, who usually offer the highest diagnostic information about oneself (Wills, 1981). Especially when objective information is absent, people make meaning of their own performance and success by comparing themselves to relevant others (Lyubomirsky and Ross, 1997). Social comparisons can occur in our daily life by both interpersonal interaction and mediated through mass communication (e.g., social media, TV shows), which both offer plentiful opportunities to gather information about other people’s actions, failures, and accomplishments (Mares and Cantor, 1992; Knobloch-Westerwick and Hastall, 2010, 2016). However, research combining the fields of media effects (especially through narratives) and social comparisons is somewhat limited, since media scholars have mainly focused on upward social comparisons (e.g., media effects related to body image; Cattarin et al., 2000; Groesz et al., 2002).

The outcome of a social comparison process (e.g., self-evaluation) is based on specific mental states, as Mussweiler (2003) describes in his *selective accessibility model* (SAM): if people are faced with the possibility to compare themselves with others, they form automatic, holistic impressions about other people based on salient features (e.g., gender, age, group affiliation). These features become a point of reference for one of the following judgments regarding self-other comparisons: (a) If the person is judged to be similar to oneself, people are more likely to consider information about themselves that is consistent with the other person. The outcome is an *assimilation effect* by adapting attributes of the person and becoming more similar. (b) If the person is considered to be dissimilar to oneself, different aspects of one’s self become more salient, which are opposite to the other person (Mussweiler, 2007; Suls and Wheeler, 2017). As a result, a *contrast effect* emerges, as recipients shift away their judgment about themselves from the other person. Contrast effects have often been studied in association with downward social comparison with less fortunate people. According to Wills (1981), people who experience threats to their self-esteem enhance their self-regard by comparing themselves downward. Likewise, cancer patients benefited from strategic downward comparisons with other less fortunate cancer patients, who they encountered in their daily life, TV shows, or newspaper articles (Wood et al., 1985). In an experiment, Mares and Cantor (1992) asked older participants to watch a portrayal about an old man, who was depicted as either unhappy and isolated or happy and socially integrated. Lonely elderly participants who watched the unhappy portrayal compared themselves downward and, as a result, felt better about themselves.

Another relevant category for downward social comparisons is group affiliation (Mastro, 2003; Mastro et al., 2008), since being part of relevant social groups is a central part of the self (cf. *social identity theory*; Tajfel and Turner, 1986). Therefore, people seek out information which favors their own social group in comparison to a relevant out-group (Harwood, 1999). For example, Mastro (2003) asked participants to read one out of two crime stories (written as a TV script) and only manipulated the name of the murderer (typical Caucasian vs. Latino name). Caucasian participants exposed to the TV script with a Latino murderer showed contrast effects by scoring higher on self-esteem measures than those who read the TV script with a Caucasian murderer. Meta-analytic data within the field of social psychology supports these findings: when a negative stereotype doubts the ability or worth of an out-group, people who belong to the in-group may experience *stereotype lift* – a performance boost that occurs when downward comparisons are made with a denigrated out-group (Walton and Cohen, 2003). This effect can also occur as a consequence of stereotypic displays in the media (for a meta-analytic review see Appel and Weber, 2017). The enhanced performance has been attributed to increased self-efficacy and decreased self-doubts as a result of negative outgroup stereotypes (Chatard et al., 2008).

### Stereotypes About (Pre-service) Teachers

Stereotypes about specific groups can be encountered in media content (Mastro and Tukachinsky, 2012). Especially entertainment media often demeans minorities, such as people with mental illness (Caputo and Rouner, 2011), overweight persons (Grabe et al., 2008), or non-Caucasians (Mastro, 2015). Regarding different professions, teachers are subject to considerable stereotyping (Carlsson and Björklund, 2010) in their professional life and during their studies, which is also evident in news and entertainment media (Swetnam, 1992). The *stereotype content model* (Fiske et al., 1999, 2002) describes stereotypes along two independent dimensions: competence and warmth. Accordingly, pre-service teachers are perceived as less competent and less motivated in their studies, but also as warm and friendly (Carlsson and Björklund, 2010).

Ihme and Möller (2015) found empirical support for the presence of these paternalistic stereotypes in a multi-study paper. First, they asked pre-service teachers about typical characteristics ascribed to their profession in an open-ended survey. Even pre-service teachers themselves believed in the incompetent, but warm stereotypes. Second, the authors asked other groups of people to rate typical characteristics of pre-service teachers, psychology, law, and computer science students on a list of competence and warmth adjectives. Results showed that pre-service teachers were perceived as significantly less competent, which includes a lack of study related motivation, compared to other fields, like psychology^1^. Furthermore, the authors found significant higher warmth ratings of pre-service teachers compared to law and computer science students, whereas there was no significant difference to psychology students.

Importantly, stereotypes like these do not require clear indications, such as open insults, to become salient. Instead, even subtle hints such as how a person is described in a news article (Gupta et al., 2014) may be sufficient in order to trigger stereotypes that are associated with a certain group as research on stereotype threat has shown (for a review of media content that triggers stereotype threat see Appel and Weber, 2017). We argue that this may also activate downward social comparisons if the person described is part of a relevant outgroup. As psychology students and pre-service teachers are perceived to be similarly warm, yet different in competence (Ihme and Möller, 2015), social comparison processes are likely to occur.

### The Current Research

The current work examines the influence of stories on the self, with (a) a special emphasis on potential contrast effects, and (b) the mediating role of transportation and experience taking during the process. In Study 1, we focused on contrast effects via downward social comparisons based on group affiliation. Accordingly, we expected contrast effects after reading a story, if recipients (psychology students) have a more distant view toward a protagonist (pre-service teacher) and the story. This distant view might be reflected by a lower degree of transportation with the main character. However, when transportation is high, we expect assimilation effects by temporarily rating oneself and behaving similar to the protagonist. To induce contrast vs. assimilation effects, we tried to manipulate transportation via reviews prior to reading the story. In Study 2, we examined contrast effects via downward social comparison based on individual differences. By adding trait measures as possible alternative explanations, we intended to clarify the relation between narrative involvement measures and potential contrast effects. In this study, we tried to manipulate experience taking by varying the narrator’s voice in two otherwise identical stories.

## Study 1

In Study 1, transportation was experimentally manipulated by presenting a brief positive (e.g., “the story was emotionally involving”) or negative review (e.g., “the story was rather unemotional”) about a story prior to reading it (Shedlosky-Shoemaker et al., 2011; Gebbers et al., 2017). Both reviews were written in a way that they might also influence experience taking. For example, the positive review about the story stated that the reader was forgetting about herself/himself by experiencing the story, as she/he felt like the protagonist herself/himself, whereas in the negative review, it was stated that the reader perceived the protagonist as strange and distant. To our knowledge, there are no studies so far which manipulated experience taking in that specific way.

It was assumed that recipients in the negative review condition compared to the control group would score lower on transportation and experience taking. Negative changes in transportation and experience taking were in turn expected to lead to (H 1a) an increase in self-reported competence ratings (but not warmth) in relation to others, (H 2a) higher learning goals ratings, and (H 3a) more time spent on an anagram task (i.e., contrast effects). Likewise, it was expected that recipients in the positive review condition compared to the control group would score higher on transportation and experience taking. Positive changes in transportation and experience taking were in turn expected to lead to (H 1b) a decrease in self-reported competence ratings (but not warmth) in relation to others, (H 2b) lower learning goals ratings, and (H 3b) less time spent on an anagram task (i.e., assimilation effects).

### Method

#### Participants

As indicated by an a-priori power analysis, for a medium direct effect (*d* = 0.50) with α = 0.05 and power = 0.80, a sample size of *N* = 159 participants is needed (one-way ANOVA with three groups). One hundred seventy-nine participants were recruited in different psychology classes at the University of Koblenz-Landau, Germany. All participants signed an informed consent in accordance with the Declaration of Helsinki before participating in the study. They were also assured that they could stop their participation without any consequences at any time. Participants received partial course credit and participated in a lottery. For the lottery, one time 30€ and seven times 10€ were raffled. The experiment was computer-based and took place in a laboratory with one to seven participants per session. Three participants had to be excluded from the sample due to technical problems. Moreover, five participants were excluded because they failed the manipulation check of the review manipulation. They could not correctly report (in an open-end text field) the valence of the review they had read as either negative or positive, indicating that they had not read the review. Another five participants were excluded as they did not correctly answer two control questions about the story, indicating that they had not read the story. Last, two participants were excluded from the final data analysis, as one indicated that the story was already known (despite the fact that the short story had been specifically written for the purpose of this study, see below), while the other did not study psychology, and thus, was not part of the in-group (see section on the stimulus text below). The final sample consisted of *N* = 164 psychology students (*n* = 131 female) with a mean age of 21.81 years (*SD* = 3.61; range: 18–49 years).

#### Material

##### Review manipulation

Both reviews were specifically written for the purpose of this study, yet structure and wording were based on previous research (Shedlosky-Shoemaker et al., 2011; Bacherle, 2015; Gebbers et al., 2017). Participants read either a negative, a positive, or no review at all of an upcoming short story (see **Supplementary Material**). Both reviews were supposed to be from an online literature community^2^ and were indicated to be written by an active and experienced community member. The reviews were comparable in word count (positive review: 218 words, negative review: 211 words) and layout design. Their main difference was the valence of the evaluation of the short story that followed. While the positive review emphasized the “intense impression of the story, which leaves the reader deeply impressed,” the negative review describes the story as “strange and leaves the reader rather unimpressed.” Moreover, there was a review on a five-star scale rated by community members at the end of both reviews (negative review: 1 star; positive review: 5 stars). After reading the review, as a manipulation check, participants were asked to summarize the main messages of the respective review in a text field. The authors thoroughly checked the open-ended answers regarding statements about the valence of the reviews. Five participant did not directly address whether the review had been positive or negative, and therefore, they were excluded from the statistical analyses.

##### Stimulus text

The experimental story (2939 words) was written for the purpose of this study and included a first-person narrator (see **Supplementary Material**). The gender of the main protagonist was not specified to avoid comparison processes based on gender differences. The story was written in a way that made it easy to imagine both a female and a male protagonist, as no gender stereotypes were addressed. It featured a pre-service teacher who struggles with his/her schoolwork, while enjoying a student’s life outside of university with partying and playing sports. The pre-service teacher attends a psychology course along with psychology students (which is common practice regarding some courses at the university where this research was conducted). While preparing for an important exam as part of this course, the protagonist struggles studying – particularly compared to fellow psychology students. As a result, he/she fails the exam. While trying to figure out reasons for this disappointment, he/she visits the professor’s office hours. The professor tells the protagonist that most students had passed the exam, mainly psychology students, while most of his or her fellow pre-service teachers had also failed. However, the protagonist gets encouraged to repeat the course the next year.

The experimental story was written in a way that typical stereotypes of pre-service teachers were not directly addressed, but rather indirectly depicted in the story. Research on group-based stereotypes revealed that even subtle cues may trigger common stereotypes (Nguyen and Ryan, 2008; Appel and Weber, 2017). We asked only psychology students (in-group) to read the story about the pre-service teacher (out-group). Accordingly, possible *downward comparisons* to prospect teachers regarding *competence* might be conceivable from the viewpoint of a psychology student, especially when they were less involved with the story and its protagonist.

##### Experience taking

In order to measure participants identification with the main character, the *Experience Taking Scale* was used (Kaufman and Libby, 2012). In our sample, the reliability of this seven-item scale was good (α = 0.90). The items (e.g., “I understood the events of the story as though I were the character in the story.”) went with a nine-point Likert scale, as in the original publication (*1 – strongly disagree; 9 – strongly agree*). The overall mean was 6.24 (*SD* = 1.68).

##### Transportation

Participants’ immersion into the story world was measured via the *Transportation Scale – Short Form* (Appel et al., 2015). In our sample, the reliability of this six-item scale was satisfactory (α = 0.78). The items (e.g., “I could picture myself in the scene of the events described in the narrative”) went with a seven-point Likert scale (*1 – not at all; 7 – very much*). The overall mean was 4.65 (*SD* = 1.15).

##### Social comparison

Participants rated their self-perceived competence (four items: competent, intelligent, diligent, and determined) and their self-perceived warmth (five items: likeable, helpful, sincere, warm, and kind) in relation to other students. Furthermore, three unrelated items (athletic, sense of humor, musical) were included as distractors (see **Supplementary Material**). The scale was adapted from the Social Comparison and Interest Scale (SCIS) by Thwaites and Dagnan (2004). As the original SCIS scale does not include the dimensions competence and warmth, we used competence and warmth adjectives based on the findings of Ihme and Möller (2015) for our scale. We took only those adjectives, which had the highest factor loadings on the two dimensions when describing pre-service teachers and psychology students (T.A. Ihme, personal communication, March 11, 2016). All items went with a bipolar ten-point Likert scale (e.g., “Compared to other students I feel… *1 – less intelligent* to *10 – more intelligent*”). The competence sub-scale showed satisfactory reliability (α = 0.76) and the overall mean was 5.98 (*SD* = 1.43). The warmth sub-scale also showed satisfactory reliability (α = 0.79) and the overall mean was 6.82 (*SD* = 1.18).

##### Learning motives

Participants’ motivation to learn was assessed with the *Scales for the Assessment of Learning and Performance Motivation School–Student Version* (SELLMO-ST; Spinath et al., 2002). It is a standardized diagnostic measure, which assesses motivational goal orientation by 31 items on a five-point Likert scale (*1 – totally disagree* to *5 – totally agree*). The SELLMO-ST contains four dimensions: learning goals (e.g., “In school I want to get new ideas.”; α = 0.71, *M* = 4.43, *SD* = 0.41), performance-approach goals (e.g., “In school I want to show that I am good at things.”; α = 0.79, *M* = 3.17, *SD* = 0.67), performance-avoidance goals (e.g., “In school I don’t want the other students to think I am stupid.”; α = 0.88, *M* = 2.36, *SD* = 0.80), and work avoidance (e.g., “In school it is important for me to do only the necessary work.”; α = 0.83, *M* = 1.93, *SD* = 0.63).

##### Anagram-solving task

As a proxy for persistency and competent behavior, we measured time spent on an anagram-solving task (Muraven et al., 1998). On the first page, participants were instructed to solve 20 anagrams. They were free to skip anagrams if they were not able to solve them. Furthermore, participants were told that they had as much time as they wanted for this task. They did not know that half of the anagrams were not solvable. In order to gather a reliable and valid measure, the entire anagram-solving task was presented on a single page, right after the introduction page, and the survey software automatically tracked the time spent on the page in the background. The overall mean was 535.66 s (*SD* = 348.73). To reduce the extreme skewness and kurtosis, time spent on anagrams was logarithmically transformed (Tabachnick and Fidell, 2007).

#### Procedure

After arriving at the laboratory, participants were welcomed and randomly assigned to one of three experimental conditions. They either read a positive (*n* = 56), a negative (*n* = 53) or no review (control baseline; *n* = 55) prior to reading the story itself. We varied the order of the material for the two review conditions versus the no review condition (control baseline). In both review conditions, participants read the review first and answered related control questions, followed by the story with two control questions. Afterwards, they answered the Experience Taking Scale and the Transportation Scale – Short Form. Next, we asked the participants to rate themselves on our adapted version of the SCIS and the SELLMO-ST. These scales were presented on separate pages due to their different rating scales (experience taking entailed a nine-point Likert scale, whereas the SELLMO-ST went with a five-point Likert scale). After reading a short instruction, participants worked on the anagram task, while the time spent on the anagram page was measured. We changed the order of the material in the no review condition to establish a true baseline. In the no review condition, participants were first asked to rate themselves on the adapted version of the SCIS and the SELLMO-ST, followed by the anagram task. Afterwards, they read the story, answered the Experience Taking scale as well as the Transportation Scale – Short Form and two control questions regarding the story. Finally, on the last page, participants in all conditions provided demographic information. Upon completion of the study, participants were debriefed.

#### Design

The experiment followed a between-subjects design with the positive vs. negative review condition as treatment and the no review condition as baseline. We propose a mediation model, with the review condition as independent variable, transportation and experience taking as mediating variables, and the SCIS – competence subscale, learning motives, and the anagram task as dependent variables.

### Results

A one-way between subjects ANOVA was conducted to compare the effect of the review manipulation on experience taking and transportation. There were no significant effects of the experimental manipulation on experience taking, *F*(2,161) = 0.29, *p* = 0.75, and on transportation, *F*(2,161) = 0.05, *p* = 0.95. Due to the unsuccessful experimental manipulation, we refrained from conducting the mediation analyses for our hypotheses. Instead, we focus on the correlations between transportation/experience taking and the dependent variables in the following paragraphs. We only report the results for both review conditions (*n* = 109), since the order of the stimulus material and measures differed between the two review conditions and the control condition.

#### Social Comparison

The SCIS – competence subscale was neither significantly correlated with experience taking, *r*(107) = -0.15, *p* = 0.12, nor with transportation, *r*(107) = -0.02, *p* = 0.82. Regarding the SCIS – warmth subscale, there was neither a significant correlation with experience taking, *r*(107) = 0.08, *p* = 0.40, nor with transportation, *r*(107) = 0.00, *p* = 0.98.

#### Learning Motives

Transportation and learning goals were significantly correlated, *r*(107) = 0.26, *p* = 0.01. However, there were no significant correlations between transportation and the other SELLMO-ST subscales; performance – approach goals, *r*(107) = 0.15, *p* = 0.11; performance – avoidance goals, *r*(107) = 0.05, *p* = 0.60; work avoidance, *r*(107) = 0.01, *p* = 0.89. Likewise, there were no significant correlations between experience taking and the SELLMO-ST subscales; learning goals, *r*(107) = 0.06, *p* = 0.55; performance – approach goals, *r*(107) = -0.03, *p* = 0.75; performance – avoidance goals, *r*(107) = 0.00, *p* = 0.97; work avoidance, *r*(107) = 0.09, *p* = 0.36.

#### Anagram-Solving Task

The correlation between experience taking and time spent on the anagram-solving task (log10 transformed) failed to reach significance, *r*(107) = 0.17, *p* = 0.08, while transportation and time spent on the anagram-solving task were significantly correlated, *r*(107) = 0.21, *p* = 0.03.

### Discussion

There was a positive correlation between transportation and time spent on the anagrams, whereas experience taking was only trend-significantly correlated to time spent on the anagrams. These results contradict our expectations, since we expected less transported participants who do not identify with the incompetent pre-service teacher in the story to contrast themselves away from the story and its main protagonist (e.g., by spending more time on the anagrams). Furthermore, the persistence to work on the anagram-solving task was not correlated to any of the other DVs. This suggests that the anagram-solving task might not be an indicator for competence self-ratings (in relation to others) or competence-related learning motives (see **Table 1**). To test an alternative explanation of this finding, we added trait conscientiousness as a broader concept in the follow-up study. Conscientiousness could be a third variable that explains the correlation between transportation/experience taking and time spent on anagrams, as the trait is related to persistence to stay on demanding tasks (Dudley et al., 2006).

**Table 1:** Correlations among variables and descriptive statistics (Study 1).

	*M (SD)*	Trans.	SCIS_Comp_	SCIS_W_	SEL_LG_	SEL_PAp_	SEL_PAv_	SEL_WA_	Anagrams
Exp.	6.23 (1.73)	0.60**	-0.15	0.08	0.06	-0.03	0.00	0.09	0.17^†^
Trans.	4.66 (1.13)		-0.02	0.00	0.26**	0.15	0.05	0.01	0.21*
SCIS_Comp_	5.98 (1.51)			0.29**	0.17	0.19*	-0.07	-0.32**	0.02
SCIS_W_	6.78 (1.24)				-0.06	-0.02	0.10	-0.08	0.01
SEL_LG_	4.42 (0.44)					0.26**	-0.02	-0.25**	0.18^†^
SEL_PAp_	3.15 (0.72)						0.46**	0.13	0.07
SEL_PAv_	2.42 (0.80)							0.18	-0.02
SEL_WA_	1.95 (0.60)								0.02


**N = 109. Exp. = E xperience Taking; Trans. = Transportation; SCIS_*Comp*_ = Social Comparison and Interest Scale - Competence Subscale; SCIS_*W*_ = Social Comparison and Interest Scale - Warmth Subscale; SEL_*LG*_ = SELLMO-ST - Learning Goals Subscale; SEL_*PAp*_ = SELLMO-ST Performance-Approach Goals Subscale; SEL_*PAv*_ = SELLMO-ST - Performance-Avoidance Goals Subscale; SEL_*WA*_ = SELLMO-ST - Work Avoidance Subscale; Anagrams = Time Spent on Anagram Task (log10 Transformed). ^†^p < 0.10, ^∗^p < 0.05, ^∗∗^p < 0.01.**

Moreover, there were no significant correlations between the narrative involvement measures and the SCIS – competence subscale. Thus, it is unclear whether or not psychology students compared themselves downward to the pre-service teacher in our study. Maybe psychology students could relate to the pre-service teacher in the story, since he or she was also a student who was taking psychology classes. In other words, group affiliation might play a less important role in perceiving a story character (at least in the context of the content of our study), than interindividual differences, such as certain personality traits in relation to the story content. Consequently, in Study 2, we did not focus on in- vs. out-group; instead, we added different measures in order to test the alternative explanation that certain personality traits influence the experience of a story with a protagonist that is described as having certain (negative) characteristics. Furthermore, since the review manipulation had no impact on transportation and experience taking in Study 1, we chose a different manipulation of story and character involvement in Study 2.

## Study 2

In Study 2, we again focused on contrast effects of stories on recipients’ selves, and payed special attention to the mediating processes of transportation and experience taking. In order to establish a causal chain, we chose another experimental approach. Instead of manipulating the conditions before reading a story (e.g., presenting a review), we manipulated specific aspects of the story itself. We manipulated the narrative voice of the story by preparing a story in which either the protagonist was the narrator of the story or the entire story was written from the viewpoint of an independent observer (first-person voice vs. third-person voice). Compared to a third-person voice, a first-person voice is expected to create a more intimate and closer connection between a recipient and a main protagonist, which strengthens experience taking (Kaufman and Libby, 2012). As in Study 1, we also included transportation as an additional measure of media involvement. However, the effect of narrative voice and related manipulations on transportation is according to Tukachinsky (2014) small to non-significant. Therefore, we included narrative engagement (Busselle and Bilandzic, 2009) as a third story-related measure. The concept of narrative engagement is strongly related to both transportation and experience taking. However, since there is little empirical evidence on the effect of the narrative voice manipulation on transportation and narrative engagement, we were reluctant to predict clear-cut effects on both measures. Therefore, we included the analyses of these effects as explorative research questions.

The experimental story was about a female negligent and very unconscientious student, who had to prepare a seminar presentation, but instead of proper preparation, she rather spent her time with a friend^3^ The story was specifically written in a way that the main protagonist was described in a rather negligent way (i.e., adjectives were used based on the personality trait conscientiousness; Ostendorf and Angleitner, 2004). Accordingly, we adapted the Social Comparison and Interest Scale (SCIS) by including these adjectives in order to capture specific media effects related to the experimental story. Moreover, we matched the study major of the protagonist of the story to our sample (i.e., media communication students).

Additionally, we included different trait measures in order to control for other third variables as alternative explanations. The broad trait measure of conscientiousness has been shown to be a valid predictor of work related behavior, like high job performance (Dudley et al., 2006), academic success (Poropat, 2009), and even the neatness of item responses in an experimental study (Paunonen and Ashton, 2001). In line with these findings, Ventura et al. (2013) found evidence that time spent on an experimental anagram and riddle task was positively correlated to a self-report measure of conscientiousness. Moreover, we controlled for participants’ study-related motives, as well as their knowledge on how to perform well in their field of study. A higher degree of similarity between recipients and protagonists (in our case low study-related motives and little knowledge how to perform well in one’s study) has been shown to block the mediating effects of experience taking (Hoeken et al., 2016).

We expected that participants who read the story with a third-person narrator would show lower levels of experience taking and transportation. Negative changes in experience taking and transportation were in turn expected to lead to contrast effects by increasing participants’ (H 1a) self-reported conscientiousness ratings in comparison to others, and (H 2b) time spent on an anagram-solving task. Likewise, we expected that participants who read the story with a first-person narrator would show more experience taking and transportation. Positive changes in experience taking and transportation were in turn expected to lead to an assimilation effect by decreasing participants’ (H 1b) self-reported conscientiousness ratings in comparison to others, and (H 2b) time spent on an anagram-solving task.

### Method

#### Participants

The total sample consisted of *N* = 81 media communication students, who were recruited in different communication studies and media psychology classes at the University of Würzburg, Germany. All participants received partial course credit. Participants signed an informed consent in accordance with the Declaration of Helsinki before the study started. Furthermore, they were informed that they could revoke their participation without any consequences at any time. The study was conducted in a laboratory with one to eight participants per session. The entire study was computer-based. Two participants had to be excluded from the analyses, because they did not correctly answer control questions regarding the experimental stories, indicating that they had not properly read the story. The final sample consisted of *N* = 79 participants (*n* = 69 female) with a mean age of 20.86 (*SD* = 1.84; range: 18–28).

#### Material

##### Manipulation of narrative voice

We manipulated the narrator’s voice similar to Kaufman and Libby (2012). Either, participants read the story written from the main character’s point of view (first-person narrator), or they read the same story from the viewpoint of a third-person narrator (see **Supplementary Material**).

##### Experimental story

The story titled “The day before” was about Tina, a negligent student of media communication, who was preparing a presentation for a course session together with a group of other students. The group was very eager to prepare a decent presentation; however, Tina was only doing as much as needed for the task ahead. Instead of thorough preparation, she preferred spending leisure time with a friend. The story (1657 words) had been specifically written for a previous study (Krause and Appel, 2017). It was slightly adapted to the current context by changing the protagonist’s field of study and university (see **Supplementary Material**).

##### Transportation

As in Study 1, participants’ immersion into the story world was measured via the Transportation Scale – Short Form (Appel et al., 2015), *M* = 4.59, *SD* = 1.10, α = 0.75.

##### Narrative engagement

In order to capture the processing of the experimental narrative in more detail, a second measure of participants’ immersion into the story world was assessed by using the Narrative Engagement scale (Busselle and Bilandzic, 2009). The twelve items went with a seven-point Likert scale; *1 – not at all; 7 – very much*. The Narrative Engagement scale (*M* = 4.80; *SD* = 0.78; α = 0.78) consists of four subscales: attentional focus (e.g., “I found my mind wandering while reading.”; *M* = 5.15; *SD* = 1.31; α = 0.86), narrative understanding (e.g., “My understanding of the characters is unclear (R).”; *M* = 6.29; *SD* = 0.72; α = 0.64), emotional engagement (e.g., “The story affected me emotionally.”; *M* = 4.20; *SD* = 1.25; α = 0.68), and narrative presence (e.g., “At times during reading, the story world was closer to me than the real world.”; *M* = 3.55; *SD* = 1.41; α = 0.81).

##### Experience taking

As in Study 1, participants’ identification with the main character was assessed with the *Experience Taking Scale* (Kaufman and Libby, 2012), *M* = 5.89, *SD* = 1.68, α = 0.90.

##### Social comparison

Similar to Study 1, participants were asked to rate themselves regarding their conscientiousness in relation to other people using ten items (DV1). Furthermore, eight other trait items were presented as distractors (see **Supplementary Material**). All items went with a ten-point bipolar Likert scale (e.g., “*1* – *less organized*” to “*10* – *more organized*”). The scale was adapted from the Social Comparison and Interest Scale (SCIS; Thwaites and Dagnan, 2004) by using adjectives from the German translation of the NEO personality inventory (Ostendorf and Angleitner, 2004). The overall mean of SCIS - conscientiousness subscale was 5.86 (*SD* = 1.41; α = 0.85).

##### Anagram-solving task

Time spent on an anagram-solving task (Muraven et al., 1998) was assessed similar to Study 1 (DV2). Participants spent on average 613.06 seconds on the task (*SD* = 554.86). As in Study 1, time spent on the anagram-solving task was logarithmically transformed (log10) in order to reduce the extreme kurtosis and skewness of this measure (Tabachnick and Fidell, 2007).

##### Control scales

Three scales were assessed in order to rule out alternative explanations (i.e., that study-related motives or the personality trait conscientiousness would influence the effect of narrative involvement measures on the DVs). First, we assessed *potential to succeed in school* (subscale of the academic belonging scale; Cook et al., 2012) with four items (e.g., “I am the kind of person that does well in my school.”), which went with a six-point Likert scale; *1 – strongly disagree* to *6 – strongly agree*. The mean for this scale was 4.29 (*SD* = 0.66; α = 0.44). Due to the low reliability of the scale, we refrained from including it in any analyses. The second scale was the *domain identification measure* (four items adapted from Smith and White, 2001, e.g., “How important is it to you to be a student of media communication?”), which went with a five-point Likert scale; *1 – not at all* to *5 – very*. The mean for this scale was 3.55 (*SD* = 0.74; α = 0.71). Third, participants were asked to rate five personality traits on the *BFI-10 scale* (Rammstedt et al., 2014) with ten items on a five-point Likert scale (*1 – disagree strongly* to *5 – agree strongly*). Thereby, participants’ self-ascribed rating of conscientiousness (two items; e.g., “I see myself as someone who does a thorough job.”) was of special interest with a mean of 3.25 (*SD* = 0.87; *r* = 0.64^4^).

#### Procedure

After arriving at the computer laboratory, participants were asked to answer the three control scales: potential to succeed in school, domain identification, and the BFI-10 personality scale. Afterwards, they were randomly assigned to one of two experimental story conditions (first-person vs. third-person narrator). After reading the story, participants were asked to answer two control questions regarding the content of the story as a manipulation check. Then, participants answered the narrative engagement and transportation scale, whereby the order of the items was randomized between and within both scales. Next, they answered the experience taking scale. Subsequently, both dependent variables were assessed, first the adapted SCIS, and second, the anagram-solving task. Finally, participants provided demographic information. Upon completion of the study, they were debriefed.

#### Design

The experiment followed a between-subjects design with the story condition as independent variable (first-person vs. third-person narrator). Like in Study 1, transportation and experience taking were included as mediating variables. The SCIS - consciousness subscale and the anagram task served as dependent variables.

### Results

Three separate *t*-tests for independent samples revealed that there was no significant effect of narrative voice on transportation, *t*(77) = -0.30, *p* = 0.77, experience taking, *t*(77) = -0.23, *p* = 0.82, or narrative engagement, *t*(77) = -0.71, *p* = 0.48, and its subscales (see **Table 2** for more information).

**Table 2:** Results of *t*-tests for independent samples between narrative voice and descriptive statistics (Study 2).

	First-Person Narrative Voice		Third-Person Narrative Voice			
				
	*M*	*SD*	*M*	*SD*	*t*	*p*
Transportation	4.56	1.14	4.63	1.07	-0.30	0.77
Narrative engagement	4.74	0.82	4.86	0.74	-0.71	0.48
NE: Attentional focus	5.18	1.40	5.12	1.24	0.21	0.83
NE: Narrative understanding	6.32	0.66	6.25	0.79	0.46	0.65
NE: Emotional engagement	4.06	1.33	4.35	1.17	-1.03	0.31
NE: Narrative presence	3.38	1.42	3.73	1.39	-1.10	0.27
Experience taking	5.84	1.87	5.93	1.48	-0.23	0.82


**N for First-Person Narrative Voice = 39. N for Third-Person Narrative Voice = 40. dfs for all t-Tests = 77.**

Again, due to the unsuccessful experimental manipulation on the narrative involvement measures, we refrained from conducting the mediation analyses for our hypotheses. Instead, the relations between variables were examined by using correlations (see **Table 3**) and stepwise multiple linear regressions analyses for each dependent variable (see **Tables 4**, **5**).

**Table 3:** Correlations among variables and descriptive statistics (Study 2).

	*M (SD)*	Dom.	BFI-10_Con_	Trans.	NE	NE-AF	NE-NU	NE-EE	NE-NP	Exp.	SCIS_rmCon_	Anagrams
Voice		-0.07	0.08	0.03	0.08	-0.02	-0.05	0.12	0.12	0.03	0.08	-0.10
Dom.	3.55 (0.74)		0.32**	0.02	-0.03	0.03	-0.19	0.04	-0.04	-0.09	0.23*	-0.06
BFI-10_Con_	3.25 (0.87)			-0.36**	-0.27*	-0.03	-0.04	-0.41**	-0.19	-0.39**	0.62**	0.04
Trans.	4.59 (1.10)				0.71**	0.26*	0.23*	0.65**	0.64**	0.58**	-0.16	0.05
NE	4.80 (0.78)					0.61**	0.48**	0.67**	0.81**	0.55**	-0.21	0.12
NE-AF	5.15 (1.31)						0.23*	-0.04	0.32**	0.12	-0.06	0.14
NE-NU	6.29 (0.72)							0.26*	0.10	0.22	0.00	0.27*
NE-EE	4.21 (1.25)								0.49**	0.56**	-0.29**	0.06
NE-NP	3.55 (1.41)									0.50**	-0.16	-0.06
Exp.	5.89 (1.68)										-0.23*	0.26*
SCIS_Con_	5.86 (1.41)											0.10


*N = 79. Voice = Narrative Voice (0 – First Person; 1 – Third Person); Dom. = Domain Identification Measure; BFI-10_*Con*_ = BFI-10 Conscientiousness Subscale; Trans. = Transportation; NE = Narrative Engagement (overall scale); NE-AF = Narrative Engagement: Attentional Focus; NE-NU = Narrative Engagement: Narrative Understanding; NE-EE = Narrative Engagement: Emotional Engagement; NE-NP = Narrative Engagement: Narrative Presence; Exp. = Experience Taking; SCIS_*Con*_ = Social Comparison and Interest Scale Conscientiousness Subscale; Anagrams = Time Spent on Anagram Task (log10 Transformed)*. *^∗^p < 0.05, ^∗∗^p < 0.01.*

**Table 4:** Hierarchical regression analysis predicting participants’ rating of conscientiousness in comparison to others (SCIS) for Study 2.

	Model 1					Model 2				
		
	*B (SE B)*	β	*t*	*p*	VIF	*B (SE B)*	β	*t*	*p*	VIF
Dom.	0.07 (0.18)	0.04	0.37	0.71	1.11	0.10 (0.20)	0.05	0.50	0.62	1.31
BFI-10-C	1.00 (0.15)	0.61	6.51	0.00	1.11	0.98 (0.19)	0.61	5.32	0.00	1.57
Trans.						0.30 (0.19)	0.24	1.62	0.11	2.58
NE-AF						-0.10 (0.12)	-0.09	-0.88	0.38	1.39
NE-NU						0.11 (0.20)	0.06	0.53	0.60	1.32
NE-EE						-0.20 (0.16)	-0.18	-1.27	0.21	2.48
NE-NP						-0.09 (0.13)	-0.09	-0.69	0.49	2.09
Exp.						0.01 (0.10)	0.02	0.14	0.89	1.79


*N = 79. Dom. = Domain Identification; BFI-10-C = Trait Conscientiousness; Trans. = Transportation; NE-AF = Narrative Engagement: Attentional Focus; NE-NU = Narrative Engagement: Narrative Understanding; NE-EE = Narrative Engagement: Emotional Engagement; NE-NP = Narrative Engagement: Narrative Presence; Exp. = Experience Taking*.

**Table 5:** Hierarchical regression analysis predicting time spent on the anagram-solving task (log_10_) for Study 2.

	Model 1					Model 2				
		
	*B (SE B)*	β	*t*	*p*	VIF	*B (SE B)*	β	*t*	*p*	VIF
Dom.	-0.04 (0.06)	-0.08	-0.65	0.52	1.11	-0.03 (0.06)	-0.06	-0.51	0.61	1.31
BFI-10-C	0.03 (0.05)	0.06	0.53	0.60	1.11	0.07 (0.05)	0.18	1.32	0.19	1.57
Trans.						-0.01 (0.05)	-0.04	-0.26	0.80	2.58
NE-AF						0.04 (0.03)	0.17	1.34	0.18	1.39
NE-NU						0.08 (0.06)	0.17	1.35	0.18	1.32
NE-EE						0.01 (0.05)	0.04	0.21	0.83	2.48
NE-NP						-0.07 (0.04)	-0.30	-1.93	0.06	2.09
Exp.						0.09 (0.03)	0.42	2.92	0.00	1.79


*N = 79. Dom. = Domain Identification; BFI-10-C = Trait Conscientiousness; Trans. = Transportation; NE-AF = Narrative Engagement: Attentional Focus; NE-NU = N rrative Engagement: Narrative Understanding; NE-EE = Narrative Engagement: Emotional Engagement; NE-NP = Narrative Engagement: Narrative Presence; Exp. = Experience Taking.*

#### Narrative Involvement

Regarding the correlation analyses, only the BFI-10 conscientiousness subscale was negatively correlated with all three narrative involvement measures, experience taking: *r*(77) = -0.39, *p* < 0.001; transportation: *r*(77) = -0.36, *p* < 0.001, and narrative engagement: *r*(77) = -0.27, *p* = 0.01 [of the four subscales only emotional engagement was significant, *r*(77) = -0.41, *p* < 0.001]. Furthermore, domain identification was not significantly correlated to any of the narrative involvement measures (for more information see **Table 3**). These results indicate that participants who were more conscientious felt less part of a story about an unconscientious student and were less likely to experience the story from the perspective of the negligent main character.

#### Social Comparison

To investigate the relation between the narrative involvement measures and participants’ self-ratings regarding their conscientiousness in relation to other people (SCIS – conscientiousness subscale, DV1), a hierarchical multiple linear regression analysis was performed. We included the control measures domain identification and trait conscientiousness (BFI-10) as first block of predictors in order to control for possible effects on the SCIS – conscientiousness subscale. The second block entailed the transportation short-scale, the narrative engagement subscales, and the experience taking scale. Tests for multicollinearity indicated an acceptable level of multicollinearity for both models (all VIFs < 3; see also **Table 4**). For Model 1, which only included the control measures as predictors, a significant regression equation was found, *F*(2,76) = 24.46, *p* < 0.001, with an adjusted *R*^2^ of 0.38. It was found that only trait conscientiousness significantly predicted participants’ ratings regarding their conscientiousness in relation to other people, β = 0.61, *p* < 0.001. Introducing the narrative involvement measures as additional predictors in Model 2, *F*(8,70) = 6.43, *p* < 0.001, with an adjusted *R*^2^ of 0.36, did not significantly add explained variance, Δ*R*^2^ = 0.03, *p* = 0.70. This finding suggests that only participants’ trait conscientiousness explained how they compare themselves to others regarding this trait, while the story had no influence on how people judge themselves in relation to others (see **Table 4** for more information).

#### Anagram-Solving Task

A second hierarchical multiple regression was conducted with time spent on the anagram-solving task (DV2). Again, the control measures were entered in the first block and the second block additionally entailed the narrative involvement measures. Tests for multicollinearity indicated an acceptable level of multicollinearity for both models (all VIFs < 3; see also **Table 5**). For Model 1, which only included domain identification and trait conscientiousness as predictors, a non-significant regression equation was found, *F*(2,76) = 0.27, *p* = 0.76, with an adjusted *R*^2^ of 0.00. Entering transportation, the narrative engagement subscales, and experience taking into the second model, *F*(8,70) = 2.25, *p* = 0.03, with an adjusted *R*^2^ of 0.11, significantly added explained variance, Δ*R*^2^ = 0.20, *p* = 0.01. Regarding the individual coefficients, only the effect of experience taking was significant, β = 0.42, *p* = 0.01. This finding indicates that experience taking and time spent on the anagrams show a positive relation, even when controlling for domain identification and trait conscientiousness (see **Table 5** for more information).

### Discussion

The findings suggest that interindividual differences in recipients’ traits in relation to the main character influence how participants experience a story. Less conscientious participants felt more like being part of the story world, and they strongly identified with the main protagonist. Indeed, character-audience similarity, respectively, familiarity with the story theme (Green, 2004) have been shown to increase transportation (Kim et al., 2016) and experience taking (Hoeken et al., 2016). A multiple regression showed a positive relationship between trait conscientiousness and participants’ self-ratings regarding their conscientiousness levels compared to others. Reasons for this finding might be that the SCIS – conscientiousness subscale is a rather trait-like measure, which might not be sensitive enough to capture state-like effects, induced temporarily through a story. However, there was still a positive relation between narrative involvement (i.e., experience taking) and how much time participants spent on the anagram-solving task. Importantly, this result, which was partly in line with Study 1, could not be explained by participants’ trait conscientiousness or domain identification.

## General Discussion

A unique feature of narratives is the power to enable us to be transported into foreign worlds (Gerrig, 1993). Moreover, being transported into a story can influence the understanding of other people (Mar and Oatley, 2008) and even how we see ourselves (Cohen, 2001; Djikic et al., 2009). The present research tried to extend previous findings on stories and the self, which focused on how recipients’ selves change in line with the story, a process called assimilation (Richter et al., 2014). Both transportation and experience taking have been shown to facilitate the influence of stories and its protagonists on recipients’ selves (Sestir and Green, 2010; Kaufman and Libby, 2012).

Yet, a story is no “magic bullet,” which automatically changes recipients’ self-perceptions in line with its content and its protagonists. Based on theoretical assumptions and previous findings, in Study 1, we expected participants, who were less involved in the narrative, to distance themselves from the story by comparing themselves downward, and thus, lifting themselves up (e.g., by being more eager to spend time on the anagram-solving task). However, the correlations between the narrative involvement measures and the dependent variables of both experiments did not support this assumption. Against our assumptions, high experience taking (in Study 2) and high transportation values (in Study 1) were both positively correlated to persistently working on a partly unsolvable anagram task. Importantly, trait conscientiousness and domain identification, which were included as a potential alternative explanation (Study 2), could not explain the effects on how much time participants spent on the anagram-solving task.

### Limitations and Future Research Directions

Despite its contribution to the literature, several limitations of this research need to be acknowledged. As we failed to find significant results regarding our hypotheses, we need to consider potential explanations why the expected results were not found. In the following, we would like to highlight three starting points that might inspire future research: (1) statistical reasons (where the studies underpowered or the design inappropriate?), (2) methodological reasons (where the manipulations or measures invalid?), or (3) theoretical reasons (is the theory invalid, and therefore, would another theory be more appropriate?).

#### Statistical Reasons

An important reason for the null-findings regarding our hypotheses is the unsuccessful manipulation of transportation and experience taking in both experiments. Indeed, findings on the effects of different approaches to manipulate transportation and experience taking are very heterogeneous between different studies, since there might be different moderating factors that influence the size of possible effects (Tukachinsky, 2014). Therefore, (a) a larger number of participants in order to avoid low-power designs and (b) assessing potential moderating factors might be beneficial for future studies. Furthermore, we used only one short story in each of the two experiments. A higher number of different experimental stories might be useful in order to show the expected effects.

#### Methodological Reasons

In contrast to our failed attempts, similar manipulations of transportation (Shedlosky-Shoemaker et al., 2011; Gebbers et al., 2017) and experience taking (Kaufman and Libby, 2012; Hoeken et al., 2016) have been successfully used before. However, other techniques to manipulate narrative involvement such as disrupting the text structure (Gnambs et al., 2014), using a non-narrative control text as baseline (Bal and Veltkamp, 2013), or giving simple instructions before reading or watching a story (e.g., “read/watch the story as if you were the character”; Sestir and Green, 2010) might have been more effective in our context.

Moreover, all our measures regarding recipients’ experience of a story were based on post exposure recall; we did not directly measure related processes in both studies. Therefore, an alternative approach to measure recipients’ experience of a story while they are reading or watching it by using psycho-physiological continuous methods, like facial electromyography, heart rate, and electrodermal activity, might be useful for future studies (Ravaja, 2004; Weber et al., 2015). Indirect or implicit measures, like the Implicit Association Test (Nosek et al., 2002) might be also valuable in order to detect more subtle temporal changes in recipients’ association between their selves and aspects of the story (Dal Cin et al., 2007; Sestir and Green, 2010; Gabriel and Young, 2011). The use of an IAT measure could be especially beneficial for assessing traits, motives, or ratings of (inferior) others compared to oneself, which might all subject to social desirability biases on explicit self-report measures (Hefner et al., 2011).

In Study 1, we expected psychology students to compare themselves downward to the pre-service teacher in the experimental story. However, future studies are recommended to control for interindividual differences in social comparison tendencies (e.g., measured via the Iowa-Netherlands Comparison Orientation Measure; INCOM; Schneider and Schupp, 2011) in order to show contrast effects. Furthermore, social comparison may be directly manipulated in an experimental approach for future studies. For example, Appel (2011) instructed participants to find dissimilarities between themselves and a stupid main protagonist. These participants (compared to participants without such an instruction) performed better in a knowledge test after reading an experimental story about a stupid protagonist, indicating a contrast effect. Future studies regarding social comparison with media personae should also take into account the salience and importance of social group categories (one’s own group vs. out groups) that could trigger social comparison processes (Trepte and Loy, 2017).

Furthermore, research on the persuasive power of stories showed that the effects of transportation on recipients beliefs (Appel and Richter, 2007) and on empathy (Bal and Veltkamp, 2013) increase over time (*absolute sleeper effect*). Likewise, possible contrast effects on participants’ selves may also appear with a time delay. Therefore, future studies on contrast effects under conditions of low transportation should also include a delayed assessment of its DVs.

#### Theoretical Reasons

In Study 1, transportation was only positively correlated to the SELLMO subscale “learning goals,” whereas both narrative involvement measures (i.e., transportation, experience taking) were not correlated to any of the other SELLMO-ST subscales. Drawing on personality traits as a potential third variable influencing this relationship, learning goals (i.e., a drive to broaden ones horizon and competences) might be linked to interindividual differences in recipients’ general tendency to become transported into a story. Therefore, future studies might also consider including a trait measure of transportability (Mazzocco et al., 2010) in order to explain assimilation vs. contrast effects.

In both studies, we found strong evidence for positive relations between high narrative involvement and participants’ perseverance to work on anagrams, even when controlling for trait consciousness and domain identification. These unexpected results could be interpreted in line with other theories and research regarding non-interactive media entertainment and well-being (Rieger et al., 2014). Indeed, a recent study demonstrated that participants who were highly involved into a narrative showed higher recovery experience and – somewhat similar to our finding – higher cognitive performance (Rieger et al., 2017). In other words, narratives enable recipients to experience a temporal self-relief (Moskalenko and Heine, 2003); it might be that even the boundaries of their selves expand while they are transported into a story (TEBOTS; Slater et al., 2014). During this process, recipients’ selves replenish, and after reading a story, they might be more energized to work on a challenging (anagram-solving) task. However, the underlying causal processes and related outcomes need to be explored more systematically in future research.

## Conclusion

The primary aim of this research was to developa better understanding of narratives’ influence how recipients see themselves compared to a story character. Furthermore, we tried to measure recipients’ motives and even their behavior after experimentally manipulating both transportation and experience taking. Going beyond previous studies on narrative effects, we did not only expect assimilation effects (changes that are in line with a story); rather, we tried to reveal contrast effects (changes that are opposite to a story). Despite the fact that our hypotheses were not supported, we are still inspired by the statement “progress occurs when existing expectations are violated” (Open Science Collaboration, 2015). Therefore, we hope that the results of this research will encourage others to future research in order to shed light on the underlying mechanisms of story reception and to advance theory of how it could influence the self in different directions.

## Data Availability

The raw data supporting the conclusions of this manuscript will be made available by the authors, without undue reservation, to any qualified researcher.

## Ethics Statement

According to institutional guidelines of the Institute of Communication and Media Psychology at the University Koblenz-Landau and the Human-Computer-Media Institute at the University of Würzburg, full ethical reviews are not required for the type of studies conducted in this research. We adhered to all ethical requirements for research with human subjects according to the German Psychological Society (DGPs) and the Declaration of Helsinki. All participants gave their informed consent in a written form.

## Author Contributions

SK and SW conceptualized and designed both the experiments. SK implemented the studies, carried out the analysis for both studies, and also wrote the manuscript with valuable input from SW.

## Conflict of Interest Statement

The authors declare that the research was conducted in the absence of any commercial or financial relationships that could be construed as a potential conflict of interest.

## Footnotes

1 It is important to note that empirical studies show *no* significant differences between pre-service teachers and other students regarding their *actual* achievement motivation and intelligence (Spinath et al., 2005).

2 https://www.leselupe.de/

3 In contrast to Study 1, we chose to use a protagonist with a specific gender, since telling a story from the view of an independent observer without directly referring to the protagonist’s gender is rather untypical for a story.

4 Spearman-Brown reliability estimate.
