# Supplementary material for: Lift Me Up by Looking Down: Social Comparison Effects of Narratives
Source: Front Psychol. 2018 Oct 18;9:1889. doi: 10.3389/fpsyg.2018.01889 (PMC6200851; doi:10.3389/fpsyg.2018.01889)
Supplement: Supplementary file 1 [file Table_1.DOCX]

Supplementary Material

Lift me up by looking down: Social comparison effects of narratives

Stefan Krause^1*^ and Silvana Weber^1^

^1^Human-Computer-Media Institute, University of Würzburg, Germany

*Correspondence:
[Stefan.krause@uni-wuerzburg.de](mailto:Stefan.krause@uni-wuerzburg.de)

# Review manipulation (Study 1)

*Positive review (original text in German)*


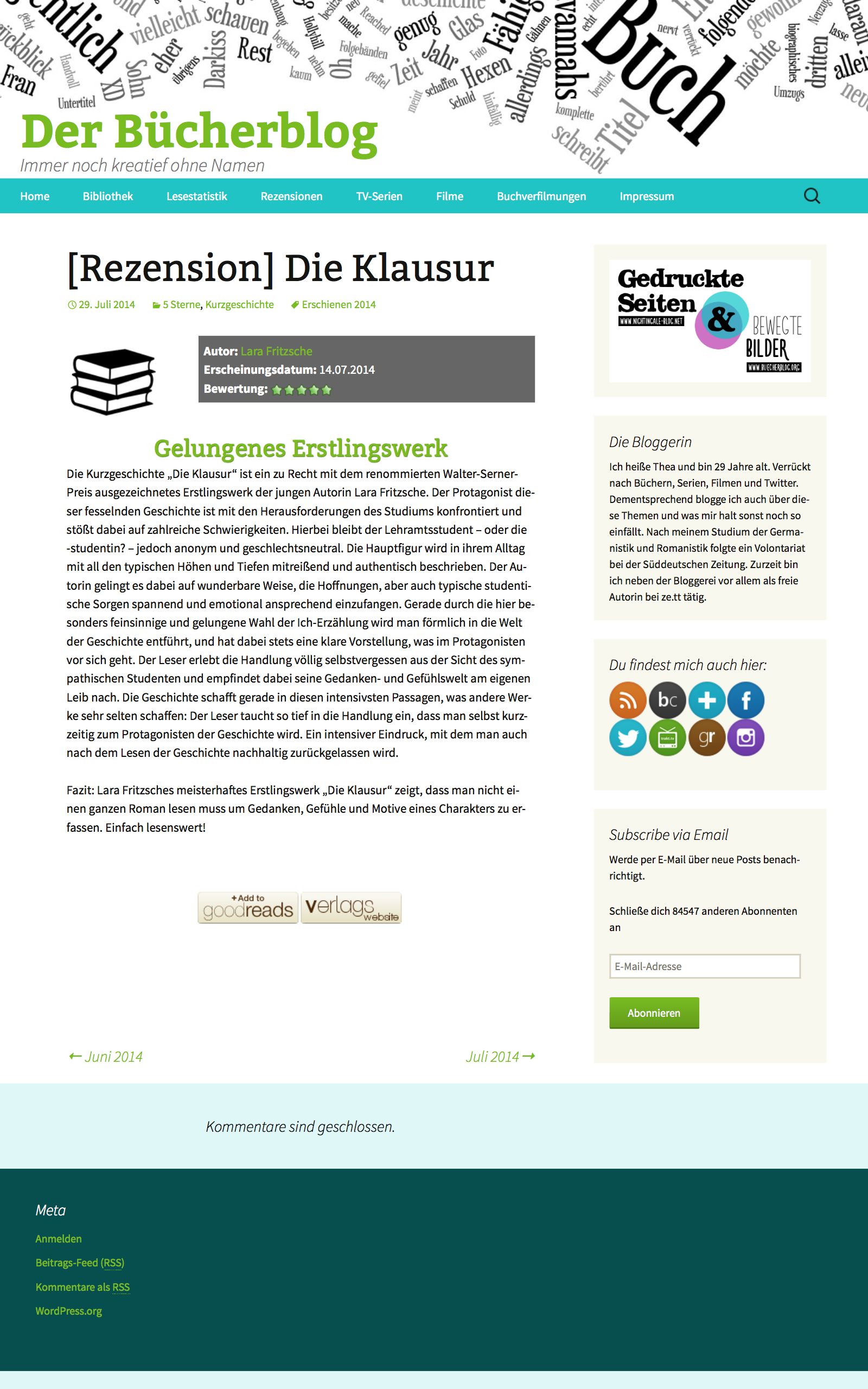


*Negative review (original text in German)*


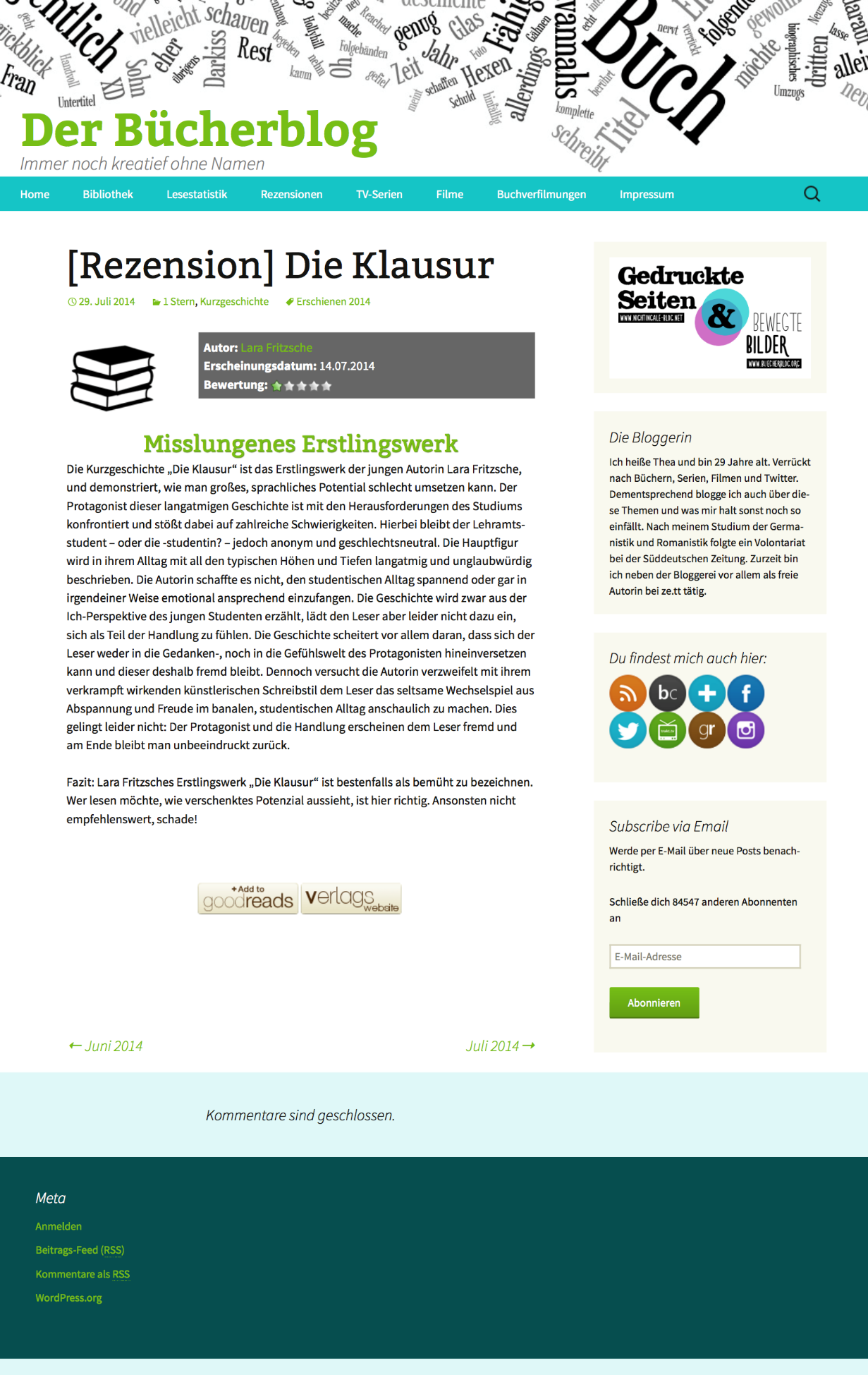


# Experimental Story for Study 1 (original text in German)

**Die Klausur**

Für mich also das Lehramtsstudium. Ganz recht, ich bereite mich auf ein Leben als künftiger Lehrer vor! Und in diesem Studium muss man nicht nur Vorlesungen zu Bildung und Sozialisation hören, sondern auch Scheine in Psychologie machen, in die Soziologie reinschnuppern und mehrere Berufspraktika absolvieren. Das finde ich toll. Warum ich diese Wahl getroffen habe? Offiziell natürlich deshalb, weil ich viel zu neugierig bin auf viele Dinge, als dass ich eine ganz klare Entscheidung hätte treffen können. „Ich habe einfach so umfassende Interessen“, hatte ich zu meinen Eltern gesagt, „und bin ja selbst auch so super vielseitig. Da kann ich mich doch nicht jetzt schon festlegen. Da würde ich mich ja total einschränken! Aber das Lehramtsstudium hält mir vieles offen, da ich zwei Hauptfächer studiere, und viele Nebenfächer belegen kann.“ Aber der wahre Grund für die Wahl dieses Fachs ist natürlich ein anderer: Ich kann mich nicht entscheiden. Ich kann mich nicht entscheiden, was ich studieren soll, und ich kann mich nicht entscheiden, welchen Beruf ich später einmal ergreifen will. Keine Ahnung, nada, niente.

Das Lehramtsstudium verschiebt diese Entscheidung um ein paar Jahre nach hinten, und das hinterlässt in mir das gute Gefühl, die eigene Spätpubertät noch eine geraume Zeit verlängern zu können. Vor allem aber gefällt mir, dass viele meiner Kommilitonen genau so denken wie ich, und ich mit meinen Unsicherheiten nicht so alleine dastehe. Irgendwie ganz anders sind da die Psychologiestudenten, die total fokussiert und zielstrebig wirken. Sie sitzen in allen Veranstaltungen zur Entwicklungs- und Sozialpsychologie immer getrennt von uns – „Psychologie für Pädagogen“ heißt das Modul in unserem Lehrplan. Tatsächlich aber sitzen wir Lehrämtler dabei in den gleichen Seminaren und Vorlesungen wie die Psychologen, die das im Hauptfach studieren, und müssen auch für dieselben Prüfungen büffeln.

Ich habe mich damals generell sehr schwer getan mit der Auswahl meines Studiums. Geschichte fand ich spannend, Jura interessierte mich, vielleicht Volkswirtschaft oder Soziologie, Journalistik fand ich auch ganz spannend, aber die Kommunikationswissenschaften hatten es mir ebenfalls angetan, und von einem Medizinstudium träumt ja sowieso fast jeder, der irgendwann mal dicke Kohle machen möchte. Die traurige Wahrheit ist aber, dass ich für viele dieser Studiengänge nicht gut genug war und der Notenschnitt auf meinem Abiturzeugnis nicht reichte. Und so verschmälerte sich meine Auswahl praktisch ganz von selbst auf einen überschaubaren Rest. Schließlich fiel meine Wahl auf das Lehramtsstudium. Ich war froh, als ich eine Universität fand, die keine Eignungstests verlangte und deren Numerus Clausus bei einem recht gnädigen Wert von 3,1 lag.

„Nun denn“, seufzte ich, als ich den Zulassungsbescheid schließlich in meinen Händen hielt. „So schwer kann‘s schon nicht werden.“ Schule, das kannte ich schließlich aus meinen eigenen bewegten Jahren in dieser Institution. Und wenn ich daran dachte, wer mich dort alles unterrichtet hatte, dann konnte so ein Lehramtsstudium doch eigentlich auch gar nicht so schwer sein, oder? Dachte ich.

★★★

Das war vor über sechs Monaten. Nun neigt sich mein erstes Semester dem Ende entgegen, und die Abschlussklausuren stehen an. Die finalen Höhepunkte. Meine erste Klausur schreibe ich im Modul „Psychologie für Pädagogen“, und während ich noch am Anfang des Studiums voller Neugierde auf das blickte, was mich in diesem Fach erwarten würde, ließ meine Motivation rasch wieder nach. Ein Blick in die müden Gesichter meiner Kommilitonen verriet mir schon in der zweiten Vorlesung, dass auch sie lieber in der wärmenden Sonne der länger werdenden Tage sitzen würden, als den drögen Monologen der alternden Dozentin Frau Dr. Meiser über frühkindliche Entwicklung zuzuhören. Und so streckten wir uns schließlich zusammen im Park auf dem weichen Rasen, sangen zum Gitarrenspiel von Kommilitonin Saskia und grillten im Schatten der grünenden Laubbäume, während die Psychos, wie wir sie nannten, still und emsig Woche für Woche in den dunstigen Bänken der Universitätssäle vor sich hinbrüteten. So vergingen die Tage des Semesters auf recht erträgliche Weise …

Doch die Klausur rückte näher. Ihr Termin schwebte über uns wie ein Damoklesschwert, und als nur noch wenige Wochen verblieben begann ich mit meinen ersten Vorbereitungen. Ich lerne jetzt erst seit ein paar Tagen. Immer wieder mal zumindest, jedenfalls versuche ich es. Aber es fällt mir schwer, mich zu konzentrieren, mich zu motivieren: Ich wache morgens um zehn Uhr auf, blinzle ein- oder zweimal in die Dunkelheit meines Zimmers, und rolle mich auf die Seite. Um 12 springe ich dann meist fluchend aus dem Bett, schiebe mir eine Pizza in den Ofen, beuge mich über meine Unterlagen und raufe meine Haare über dem drögen Akademikersprech und unverständlichen Diagrammen, verabrede mich lieber mit Saskia zum Slacklinen oder mit Michael zum Basketballspielen. Wenn ich abends heimkomme, plaudere ich noch eine Weile mit meinen Mitbewohnern über einem leckeren Merlot. „Mach einen Lernplan“, schlägt meine Mitbewohnerin Lisa vor, als ich mich bei einer dieser Gelegenheiten über meine Motivationsprobleme auskotze. „Vielleicht hilft dir das, den Stoff in kleinen, leicht verständlichen Abschnitten vorzubereiten.“

Mehrmals verwerfe ich den eher halbherzigen Entwurf meines Lernplans, als sich irgendwann eine chaotische Abfolge von Tagen ausgelassenen Vergnügens und Stunden panikgetrieben Lernens einstellt. Immer, wenn ich anfange, die Absätze in den dicken Lehrbüchern mehrmals lesen zu müssen oder meine eigenen Aufzeichnungen plötzlich keinen Sinn mehr ergeben, greife ich zum Handy und rufe einen meiner Kommilitonen an. „Um mich abzulenken“, wie ich sage, „zum Sackenlassen. Denn auch Wissen muss schließlich arbeiten.“ Leider passiert das ziemlich oft. Aber ich lebe nur ein Mal, sage ich mir immer wieder mit grimmiger Entschlossenheit, und möchte später nicht bereuen müssen, dass ich die letzten Tage meiner dahinwelkenden Jugend hinter den grauen Mauern der Universitätsbibliothek in der staubtrockenen, eintönigen Gesellschaft langweiliger Bücher verbracht habe. Langweilen kann ich mich, wenn ich tot bin.

So fliegen die Tage trotz aller Lernschwierigkeiten dahin. Ich gehe zur Uni, wenn ich muss. Ich habe einen Stundenplan und arbeite ihn ab. Zwänge sind besser als diese Unsicherheit, denke ich, und eigentlich lerne ich auch nur aus dem Zwang, keine schlechte Note schreiben zu wollen. Natürlich ist die „Psychologie für Pädagogen“ interessant, aber so interessant, dass ich gern wochenlang büffle, nun auch wieder nicht. Natürlich macht die Uni Spaß, aber mehr Spaß macht eigentlich der Kosmos drum herum. Das ändert sich auch nicht, als der große Klausurtermin plötzlich nur noch wenige Tage entfernt ist, und ich mittlerweile sogar vermeide, auf den großen Kalender in unserer Küche zu schauen. Aber befreien kann ich mich von der zunehmenden Panik nicht. Ich weiß, dass ich bisher nicht gerade viel gelernt habe, obwohl ich es eigentlich hätte tun sollen. Ich verfalle immer wieder in ziellose, panische Überaktivität, wühle, getrieben von Angst, durch meine Ordner, erschrecke über der schier unüberblickbaren Menge an Artikeln und Präsentationen, brülle dann meinen Laptop an: „Wer soll das denn alles lernen? Und wann?“ Ich verzweifle noch etwas mehr, schreibe in WhatsApp hektische Nachrichten an meine Seminarskollegen in unserer Lerngruppe *Psycho-Zeugs fürs Lehramt* und schicke stumme Stoßgebete aus in der Hoffnung, dass jemand mir mit einfachen Worten aushelfen oder mit guten Erklärungen die Knoten in meinem Verstehen glätten möge. Doch die sind ebenfalls alle ratlos. Michael schickt mir nur drei Fragezeichen als Antwort, Saskia einen traurigen Smiley. Allmählich dämmert mir, dass es vielleicht keine so gute Idee war, das ganze Semester über im Seminar zu schlafen und die Vorlesungen durch Parkbesuche zu ersetzen. Doch ich habe keine Zeit für Reue, stürze mich wieder ins Lernen, aber ich habe überhaupt keinen Plan davon, was ich eigentlich vorbereiten soll. Ich weiß nicht, was verlangt wird, ich weiß nicht, wie viel, und verstehen tue ich das sowieso alles nicht. *Zu viel, viel zu viel! Viel zu komplex, viel zu abstrakt. Wer schreibt denn so was? Wer liest den Mist? Und wer braucht das? Ich schaffe es nicht, schaffe es nicht …*

Mit Wucht stoße ich das dicke „Lehrbuch der Entwicklungspsychologie“ von mir und lasse mich rücklings aufs Bett fallen. Ich schließe die Augen. *Keine Chance … Ich schaff‘s nicht.* Unter der Last dieser Erkenntnis schwinden mir die Sinne. Ich versinke in einem unruhigen, traumlosen Schlaf …

★★★

Es ist soweit. D-Day! Mit einem Schreibblock, zwei sorgfältig gespitzten Bleistiften und einem Füller bewaffnet, begebe ich mich zum Seminarraum, in dem die Klausur geschrieben werden soll. Als ich den Kopf hebe und vorsichtig durch den Türrahmen in den engen, muffigen Raum schiele, begegne ich dem ausdruckslosen Blick eines Mädchens aus meinem Psychologie-Seminar. Wie heißt die noch mal? Clementine oder Charlie oder so. Jedenfalls eine von diesen eifrigen Psychologiestudentinnen, die nichts Besseres zu tun haben, als versonnen auf die komplizierten Diagramme in ihren Lehrbüchern zu starren und ganze Sachbücher auswendig zu lernen. Wahrscheinlich mitsamt Impressum und Literaturverzeichnis. Wie strebsam sie und ihre Gruppe abgeklärter Psychologen immer waren und wie aufmerksam sie zuhörten, wenn die Professorin etwas vortrug… puh. Niemand, der klar bei Verstand ist, hört 90 Minuten lang interessiert dem Geschwafel einer alten Knackerin zu, die im Vorbeigehen eine Dunstwolke aus Mottenkugeln hinterlässt. Ich schüttle den Kopf und gehe an dem Mädchen vorüber, meine schwarze Stofftasche streift den Ärmel ihrer Bluse. *Irgendwie cool ist sie trotzdem*, denke ich fast ein bisschen neidisch. *Und feiern kann sie auch.* Jedenfalls habe ich sie bei fast jeder Fachschaftsparty mit ihren Psychologen-Freundinnen gesehen. Nur gegen Ende des Semesters nicht mehr, als sowieso die meisten standhaften Partygänger kalte Füße bekamen und sich lieber dem Lernen zuwandten als dem rauschenden Leben. *Spießer!*

Nervös wippe ich mit dem Fuß. Vor mir, in der Mitte des Pults, liegt umgedreht ein unscheinbarer Bogen bedruckten Papiers. Ich starre darauf hinab. Meine Finger kneten nervös an seinen Ecken und Kanten, während ich an die Stufen der Moralentwicklung nach Kohlberg – oder hieß er Kohlmann? – denke, über die Entwicklungsstufen von Pia-Dingsbums, dieser Franzose – oder ein Schweizer? – grüble, und ich plötzlich nicht mehr unterscheiden kann, welche Theorie nun was besagt, und in welchem Alter nun was passiert?

Alles, was ich notdürftig gelernt habe in den vergangenen Wochen, ist plötzlich weg! Alles, wirklich alles.

Das Blut schießt mir in den Kopf, meine Adern pulsieren, mir wird heiß, und ich zittere. Ich schlucke schwer, blicke immer wieder auf den unschuldig daliegenden Klausurbogen, als könnte mein Starren allein ihn zum Verschwinden bringen. Ich streiche mir über das Gesicht, es fühlt sich nass an, aber das ist vielleicht weniger schlimm als das Gefühl, Melkfett abzusondern.

Ich versuche tief durchzuatmen, ein, aus, ein, aus, doch irgendwie klappt es nicht so richtig. Meine Atmung ist flach und schnell. Irgendwie leide ich jetzt unter einer Form von lerntraumatischer Kurzzeitamnesie – oder konnte ich mir schon vorher nichts merken? Ich verlasse den Raum, renne auf die Toilette, reiße die Tür zur zweiten Kabine von links auf, drehe den Schlüsselknauf von innen herum und lehne mich an die Tür. Ich atme immer schneller, ich habe alles vergessen, ich halte dem Druck nicht stand. Das blaue Antifixerlicht in der Toilette flackert unerträglich. Schrill kreischt eine Stimme zwischen meinen Schläfen, dass ich nichts verstanden hätte, dass ich gar nichts könne, nicht mal das Wenige, von dem ich dachte, dass ich es nach einmaligem Querlesen zumindest so ein wenig durchdrungen hätte. Nichts ergibt mehr einen Sinn. Mir fallen die ganzen wichtigen Begriffe nicht mehr ein, die komplizierten Modelle von Herrn Soundso und Doktor Wiehießernoch. Wäre das eine mündliche Prüfung, könnte ich mich vielleicht irgendwie retten. „Door in the face“ nennt Kommilitone Michael dieses Vorgehen, den Professor möglichst von der ersten Minute an mit einem Schwall von Wörtern zu übergießen. Absolute Souveränität bei völliger Ahnungslosigkeit oder so ähnlich. Ja, manchmal klappt das, und, ja, es habe ihn auch schon mal vorm Durchfallen bewahrt, meinte er. Aber hier und jetzt ist das keine Option für mich, keine Ausflucht.

Ich schaue auf die Uhr. Kurz nach zwei. In spätestens zehn Minuten teilt Frau Dr. Meiser die Klausuren aus. Ich atme tief durch, zwinge meine wirbelnden Gedanken zur Ruhe. Dann drehe mich um, schiebe mich langsam und mit bebenden Beinen Schritt für Schritt in den Seminarraum zurück. Mit gesenktem Kopf betrete ich die Höhle des Löwen.

★★★

Zwei Wochen später sitze ich auf einem braunen Kunstfaserteppich vor einem Raum mit geschlossener Tür. Neben mir sitzt Michael, neben ihm haben sich Saskia und noch vier, fünf, nein, sechs Leute aufgereiht, die ich flüchtig aus meinen Seminaren kenne. Wir warten auf Frau Dr. Meiser, die „Mutter der Psychologie“, die „Herrin der Zensuren“. „Sprechstunde mittwochs, 14 bis 15 Uhr“, ist im Vorlesungsverzeichnis hinter ihrem Namen vermerkt. Jetzt ist es fünf nach zwei, doch die Tür von Frau Dr. Meisers Büro ist verschlossen.

Ich will in ihre Sprechstunde gehen, weil auf dem Pinnbrett im Flur des Instituts meine Matrikelnummer angeschrieben steht, direkt unter der Überschrift „Klausur Entwicklungspsychologie [Veranst.-Nr.: 3678]: nicht bestanden“. Nun will ich Frau Dr. Meiser persönlich danach fragen, „zur Rede stellen“, so hatte ich vor Lisa noch groß getönt. Wahrscheinlich werde ich aber bloß höflich nachfragen und mir danach einen netten Ratschlag für die Nachschreibeklausur geben lassen.

„Ich muss um fünfzehn Uhr zu meiner Vorlesung“, schrillt plötzlich eine Stimme durch den Flur. Köpfe wirbeln herum. „Wir haben eine halbe Stunde Sprechstunde, hier sitzen acht Leute. Bitte fassen Sie sich kurz, wir haben für jeden drei Minuten. Wer ist der Erste?“
Ich gehe hinter ihr durch die Tür, Frau Dr. Meiser wühlt mit einer Hand in ihrer Ablage herum, mit der anderen zeigt sie auf einen dreibeinigen Schemel, der vor ihrem Schreibtisch steht. Offenbar soll ich mich da hinsetzen. Ich nehme Platz, der Schemel wackelt, und irgendwie komme ich mir vor wie ein Erstklässler, der zu seiner Lehrerin aufblickt. Frau Dr. Meiser beginnt das Gespräch mit einer Frage: „Ihr Name?“

Ich sage artig meinen Namen und erwähne, dass ich in ihrem Entwicklungspsychologiekurs gewesen bin, dass ich die Klausur mitgeschrieben habe und dass ich leider Gottes durchgefallen bin.

„Das ist sehr schade, und was führt Sie dann zu mir?“

„Ich wollte mal sehen, was ich falsch gemacht habe, und fragen, ob ich eine Wiederholungsklausur schreiben kann.“

Frau Dr. Meiser wühlt in ihrem Stapel herum, und murmelt dabei: „Hätte ich bloß die Noten erst in den Semesterferien aufgehängt, dann käme jetzt niemand auf die Idee, in die Sprechstunde zu kommen und rumzumeckern.“ Dann zieht sie meine Arbeit heraus.

„Hier“, sagt sie und reicht sie mir herüber, „Sie sind zwei Punkte an einer Vier vorbei und das, obwohl die Klausur insgesamt recht gut ausgefallen ist. Mir ist aufgefallen, dass in dieser Prüfung fast ausschließlich Lehramtsstudenten durchgefallen sind. Ich weiß wirklich nicht, was bei Ihrer Gruppe schiefgelaufen sein könnte. Haben Sie dafür vielleicht eine Erklärung?“ Sie sieht mich fragend an.

Ich nehme den Zettel und blicke darauf, einige wenige Häkchen finden sich auf dem Blatt, dafür aber sehr viele Fs für Fehler oder falsch oder fehlend oder was auch immer.

„Hmm, nun. Ich hätte mich vielleicht etwas besser vorbereiten müssen, war da vielleicht ein wenig … nachlässig“, sage ich vorsichtig und denke an die vielen Studentenpartys und Parkbesuche, das Slacklinen mit Saskia und Basketball spielen mit Michael. Dann dringen jedoch die vielen Male in mein Bewusstsein, als ich mit leerem Blick vor meinem Schreibtisch saß, auf die Unmengen von Wörtern gestarrt habe, die für mich einfach keinen Sinn ergeben wollten, die Berge von Begriffen, Definitionen, Experimenten, Namen …

„… Aber die Klausur war auch wirklich, wirklich sehr schwer“, beende ich den Satz. „Und … ja, es war auch ziemlich viel Stoff. Fast zu viel, wie ich finde. Kann ich vielleicht eine Nachschreibeklausur schreiben?“, frage ich zaghaft.

Sie blickt mich lange an, mustert mich nachdenklich durch die dicken Gläser ihrer Brille. „Kommen Sie doch einfach in zwei Semestern wieder in meinen Kurs und probieren es noch mal“, schlägt sie vor und nimmt meine Klausur wieder entgegen. „Sie haben sehr viele Lücken gehabt und einige Begriffe falsch verwendet. Es ist aber wichtig, dass Sie fundierte Kenntnisse von dem haben, was hier geprüft wird, denn schließlich sollen Sie das Wissen doch später auch bei Ihrer Arbeit mit Menschen anwenden. Wir prüfen Sie ja nicht, um Sie zu ärgern.“ Sie lächelt freundlich.

Ich sacke innerlich etwas zusammen, sage ihr, dass es doch eine Nachschreibeklausur geben soll und dass ich lieber nachschreiben würde, als den Kurs nochmals zu belegen, und dass es doch ohnehin nur ein Nebenfach für mich sei und der Fokus meines Studiums ganz woanders liege und und und…

Sie schüttelt den Kopf, als ich verstumme, versuche, sie mit meinen Augen allein zu beschwören. Mit ruhiger, fast sanfter Stimme erwidert sie: „Ich kann ihren Ärger verstehen. Aber leider kann ich in dieser Sache nichts machen. Ich weiß, dass Sie Lehramtsstudent sind und nicht Psychologiestudent, aber es gelten für Sie dieselben Anforderungen und Bedingungen wie für alle anderen Studenten in meinem Kurs auch. Das habe ich Ihnen allen zu Beginn des Semesters gesagt. Es liegt ganz in Ihren Händen, wie Sie sich vorbereiten und welche Leistung Sie erbringen.“

Sie blickt mich bedauernd über den schwarzen Rand ihrer großen, runden Brillengläser an und ich greife mit einer Hand nach meinem Rucksack. Dann erhebe ich mich und murmle: „Alles klar. Sie haben wahrscheinlich recht“, seufze ich.

„Kommen Sie in einem Jahr wieder“, sagt sie, diesmal munterer, „denn dann werde ich das Seminar zusätzlich zur Vorlesung wahrscheinlich noch einmal für Sie und Ihre Mitstudenten gesondert anbieten! Dass so viele durchgefallen sind, muss ja einen Grund haben. Es kann durchaus sein, dass Sie mit dem Stoff überfordert waren, wie Sie sagen, und dass es so viele von Ihren Kommilitonen ebenfalls getroffen hat, kommt ja schließlich nicht von ungefähr.“ Ich kratze mich am Kopf, grinse sie schief an. Sie lächelt zuversichtlich.

„Ich drücke Ihnen fest die Daumen für das nächste Mal. Sie schaffen das. Ganz bestimmt. Wenn Sie diesmal gut mitarbeiten und sich ausreichend Zeit für die Vorbereitung nehmen, steht einer guten Note nichts mehr im Wege. Sie werden sehen!“

Ich nicke, wende mich ab, und als ich in den Flur hinaustrete, verdeckt die zufallende Tür den Blick ins kleine, kahle Arbeitszimmer, das schmale Fenster, die Stapel der Klausurbögen und Frau Dr. Meisers wohlwollendes Lächeln.

# Experimental stories for Study 2 (original text in German)

*First-person narrator*

**Der Tag davor**

Ein Geräusch dringt an mein Ohr. Schrill und beständig sticht es in mein schlaftrunkenes Bewusstsein wie die Nadel einer Maschine in widerstrebenden Stoff. Ich runzle die Stirn und versuche, es zu verbannen, aus meiner Wahrnehmung zu verdrängen wie die Gedanken an das Unvermeidbare, das mir nun bevorsteht. Ich schnaube leise. „Nein, noch 5 Minuten“, denke ich. Meine Hand tastet suchend in Richtung des penetranten Geräuschs. Meine Finger strecken sich, schieben sich über die kühle Oberfläche des Plastikgehäuses meines Funkweckers und suchen nach der schlafbringenden Erlösung. „5 Minuten... nur noch...“, sind die einzig klaren Gedanken, die ich fassen kann. Der Wecker gibt nach, das Geräusch verstummt. „...5 Minuten“, denke ich und seufze zufrieden.

Widerstrebend öffnen sich meine Lider erneut. Stumm starre ich durch das schattige, trübe Zwielicht zur Decke. Ich gähne. Sekunden verstreichen, ehe ich einen Blick auf die digitale Anzeige neben mir riskiere und mich selbst murren höre: „Nun gut. Dann eben 20 Minuten. Passt.“, denke ich mir. Ich strecke mich genüsslich. Dann bringen meine Ellenbogen meinen protestierenden Oberkörper langsam in eine aufrechtere Lage. Doch mein Kopf sackt in den Nacken. *Nein*, denke ich gequält. *Nein. Keine Lust. Ich habe einfach... keine Lust*. Ich seufze und verharre einen Moment in dieser Haltung. *Muss wohl. Bringt ja nichts,* ächze ich leise vor mich hin.

Träge hieven unwillige Arme und Beine meinen bleiernen Körper über die Bettkante. Eine Hand greift widerstrebend nach achtlos über die Stuhllehne geworfenen Kleidungsstücken, die andere zerrt lustlos am Zugseil des Rollladens. Unter größtem Protest bahnen meine Füße sich schließlich einen Weg über den Boden, treten dabei nachlässig verstreute Gegenstände und Papiere über den Teppich und tragen mich Richtung Bad.

„Was liegt an, Tina?“, frage ich brummend mein Gesicht in der fleckigen Spiegelfläche. „Nein, sag's nicht. Irgendwas mit Uni.“ Ich wende mich von meinem Spiegelbild ab.

*Keine Lust*, denke ich, als meine Hände die Hose über meine Beine streifen. *Keine Lust*, während ich die Falten im Shirt glatt streiche. Mein Blick begegnet mir im Spiegel. Ich versuche, meine Gedanken zu sammeln, mich zu konzentrieren auf... *Keine Lust.*

Seufzend kehre ich zurück und verharre im Rahmen der Zimmertür. Skeptisch wandert mein Blick über Berge von Gegenständen, die sicherlich ihren angestammten Platz hätten, wenn ich nur nach einem für sie suchen würde… Eilig verstaute und aus Ordnern gefallene Aufzeichnungen lugen hinter Schränken und Kommoden hervor. Stifte liegen auf, hinter und unter dem Schreibtisch, umringt von haufenförmigen Ansammlungen aus unsortierten Klamotten. Mein Blick fällt auf mehrere verstreut liegende Papiere. Eines wurde vom Wind aufgewirbelt und auf den kleinen Zierkaktus gespießt, der vertrocknet in krustiger Blumenerde auf dem Beistelltisch steht. Den vielen Formeln nach zu urteilen könnte es Statistik sein… trockener geht es kaum. Innerlich sacke ich ein wenig zusammen.

Dann gebe ich mir einen Ruck. Ich raffe mich auf, die Papiere vom Boden zu sammeln und versuche, sie zu ordnen. Media Richness, Reduced Social Cues, SIDE – die Aufzeichnungen sind mit Begriffen gefüllt, die mir vage bekannt vorkommen, aber nie wirklich in meinem Kopf abgespeichert wurden. Irgendwas mit Medien halt. Ich habe Mühe, die einzelnen Seiten in eine stimmige Reihenfolge zu bringen. Mehrfach muss ich sie hin- und herwenden, nebeneinander legen und angestrengt die Zeilen an ihrem Anfang und Ende vergleichen, bis ich schließlich aufgebe und sie nach einem müden Blick auf die Uhr eher willkürlich in meinen Ordner hefte.

Auf dem Weg zum Gruppentreffen grüble ich lustlos über unsere letzte Sitzung nach. Über das Referat zur Media Equation, das wir vorbereiten müssen, und den unerträglich straffen und kleinschrittigen Zeitplan, den ein Kommilitone vorgeschlagen und für uns alle vorbereitet hat. „Gute Idee, findet ihr nicht“, fragte er stolz in die Runde. Ich wölbte die Brauen, verzichtete aber auf eine Antwort. Wie kann man so unentspannt sein? Es gibt Wichtigeres als Bücher, Skripte und ständige Lerntreffen. Wissen diese Leute überhaupt, wie man lebt?

Vor einer Haustür in der Sanderau bleibe ich stehen und hebe den Finger zum Klingelknopf. Prompt wird die Tür aufgerissen, und Max, der Kommilitone mit dem straffen Zeitplan, funkelt mich herausfordernd an:

„Du kommst zu spät“, schnauzt Max. „Wir warten schon seit einer halben Stunde auf dich, du Faulpelz! Hättest dich wenigstens melden können.“

Ich hebe beschwichtigend die Hände. „Hey, hey! Sorry. Bin ja jetzt hier.“

Mit dem Kopf deutet er ruckartig in die Küche hinter sich. Ich folge ihm zur Gruppe, die schweigend auseinanderrückt, um mir Platz zu machen. Jemand sieht mich prüfend an:

„Verpennt?“

Ich schnaufe abfällig. „Kommt vor.“

„Hast du deinen Teil vorbereitet?“, fragt Max.

Ich denke nach. Nur schwach entsinne ich mich der Inhalte, die ich gestern noch vor dem Zubettgehen überflogen hatte. Media Equation bedeutet, dass Menschen dazu neigen, Computer wie reale Personen zu behandeln. *Ziemlich selbsterklärend*, beschloss ich, und verzichtete deshalb darauf, mir irgendwelche Notizen zu machen.

„Ein wenig“, erwidere ich ausweichend, ohne zu überlegen. „Das Wichtigste eben.“ Insgeheim hoffe ich, dass mir weitere Nachfragen erspart bleiben.

Das Treffen verläuft so, wie ich es erwartet hatte: Sinnloses Beisammensitzen mehr oder weniger motivierter Menschen, die sich über Aufteilung, Reihenfolge und Inhalt der Referatsteile zanken. Ich halte mich zurück, besonders viel weiß ich ohnehin nicht. Während die anderen miteinander reden, blättere ich lustlos durch meine unsortierten Unterlagen. *Kreative Ordnung in deinem Ordner*, denke ich selbstironisch. Dann erzählt jemand, auf welch einfallsreiche Weise er seinen Teil vortragen wolle und verweist dabei auf eine Textstelle, die ich gar nicht finden kann. *Hm, blöd. Wohl daheim vergessen. Egal, bis morgen wird sich bestimmt alles finden*. *Habe mich allerdings schon mehrmals bei so etwas verschätzt...* Ob ich meinen Teil versuchsweise vortragen könne, fragt plötzlich der straff terminierte Kollege Max. „Nein“, erwidere ich und verdrehe innerlich die Augen. „Aber ich werde ihn bis zur Präsentation morgen fertig haben.“ *Das heißt dann, wenn er wirklich wichtig ist*, füge ich stumm hinzu.

Die Gruppe beschließt eine endgültige Reihenfolge und diskutiert ein letztes Mal die konkreten Inhalte. Ob ich mit dem Ergebnis zufrieden sei, werde ich gefragt. Ich hebe die Schultern, nicke dann. Eigentlich interessiert mich das Ganze nicht sonderlich. Wird schon schiefgehen.

Wieder zu Hause, schleudere ich den Rucksack zu Boden und hole mein Handy hervor. Ich lese die Nachricht eines Freundes, der mich fragt, ob ich Lust hätte, mit ihm einen Film zu schauen. Ich zögere. Eigentlich sollte ich an meiner Präsentation arbeiten. Ich bestehe das Seminar nur, wenn ich eine halbwegs passable Präsentation abliefere. Ohne Referat kein bestandenes Seminar, also auch kein Notenbonus. Aber den Notenbonus könnte ich wirklich gut gebrauchen. Unschlüssig lasse ich das Handy sinken und blicke auf den Rucksack mit meinen Referatsmaterialien. *Erst mal etwas essen*, denke ich. *Danach kann ich immer noch entscheiden, was ich mache.*

Während der Mahlzeit vibriert mein Handy. Eine Nachricht vom eifrigen Kommilitonen Max: Er erinnert uns als Gruppe unter Einsatz einiger überflüssiger Satzzeichen daran, dass wir für morgen unbedingt und unter allen Umständen „an alles Wichtige denken müssen, das ist wirklich, wirklich wichtig, sonst stehen wir echt doof da!!!!!“ Ich grinse. *Fünf Ausrufungszeichen. Rufzeichen sind keine Rudeltiere… Wissen diese Leute, wie man lebt?*

Ich stapele das Geschirr übereinander und schiebe es beiseite. In meinem Zimmer lasse ich mich aufs Bett sinken. Zum Rhythmus meiner trommelnden Finger erwäge ich, ob ich zu meinem Freund gehen und den Film schauen oder lieber den Referatsteil vorbereiten sollte. Mehrere Augenblicke lang starre ich in die Leere vor mir. Die Entscheidung fällt mir schwer. *Ach*, denke ich mir, d*er Tag ist noch lang*, und springe auf. Ich nehme die Jacke vom Haken. *Und für die Arbeit brauche ich Motivation, positive Energie,* denke ich mir beschwingt. *Die kommt nicht von uninspiriertem Rumhängen über blöden Büchern!* Hinter mir fällt die Tür ins Schloss, als ich mich gutgelaunt auf den Weg zu meinem Freund mache.

Früh am Morgen kehre ich mit dröhnendem Schädel zur Wohnung zurück. Vorsichtig öffne ich die Haustür, schleiche zu meinem Zimmer und schlüpfe aus den Schuhen. Meine Mitbewohnerin reiht ihre stets akkurat entlang der Wand auf, ich hingegen verteile meine in überschaubarem Durcheinander vor meiner Zimmertür. Mit bloßen Zehen taste ich mich über den kaum sichtbaren Teppich zu meinem Bett. Dort ziehe ich mich aus, lasse meinen Blick kurz durch den völlig überfüllten Raum gleiten und verstaue meine Klamotten schließlich achselzuckend als Knäuel in einem schmalen Spalt zwischen der Schrank- und Zimmerwand. *Hat Platz und Halt*, denke ich.

Erschöpft sinke ich aufs Laken, presse eine Hand gegen die Schläfe und schließe die Augen. *Was für ein geiler Abend! Besser hätte er nicht laufen können. Vielleicht wiederhole ich das morgen, direkt nach...* Plötzlich reiße ich die Augen auf. Das Referat! Mein Vortrag!

Ich schnelle hoch. Der Rucksack, wo ist er? Ich knipse das Licht an, starre panisch auf das gewohnte Chaos, versuche, mich mit Blicken allein zu orientieren. Keine Chance! Hastig wühle ich mich durch Berge getragener Hosen, Shirts, diverser Papiere und sonstigen Zeugs, das sich irgendwie, irgendwo in das vertraute Durcheinander fügt. *Verdammt. Verdammt! Warum liegt denn alles durcheinander? Idiot! Du wolltest gestern schon aufräumen!* Wieder und wieder halte ich inne, versuche Ordnung zu schaffen, schaufele dabei jedoch bloß Gegenstände von einem Haufen auf den nächsten. Ich schlucke schwer. *Das ist doch nicht möglich!* *Wo habe ich das Ding hingestellt?* Ich drehe mich ratlos im Kreis, fühle mich aufgeschmissen, irgendwie überrumpelt. Plötzlich ertastet eine Hand unter der achtlos hingeworfenen Jacke die Tragelasche des Rucksacks. Ich schließe die Augen, stoße erleichtert die Luft aus. Endlich! *Verfluchtes Referat!* Ich hole die mittlerweile stark geknitterten Seiten hervor, kneife die Augen zusammen und überfliege mit trübem Blick eilig die Absätze. Konzentriert versuche ich, mir Inhalte zu merken, kaue nachdenklich auf meiner Lippe und starre gelegentlich an die Wand, darum bemüht, mir Fetzen des Geschriebenen einzuprägen. *Mann,* z*iemlich viel*. *Was für ein Stress*, grübele ich müde.

Ich halte inne.

Und lasse die Papiere sinken.

*Aber schon alles irgendwie selbsterklärend, oder?,* denke ich. *Wozu brauche ich Notizen? Wie schwer kann das schon sein? Bin ich eben spontan und denke mir den Text aus, wenn es soweit ist,* überlege ich. *Stand-Up-Science. Kommt eh besser als diese auswendig gelernte Leierei.* *Passt. Wird schon schiefgehen*, sporne ich mich selbst an.

*Third-person narrator*

**Der Tag davor**

Ein Geräusch dringt an ihr Ohr. Schrill und beständig sticht es in ihr schlaftrunkenes Bewusstsein wie die Nadel einer Maschine in widerstrebenden Stoff. Sie runzelt die Stirn und versucht, es zu verbannen, aus ihrer Wahrnehmung zu verdrängen wie die Gedanken an das Unvermeidbare, das ihr nun bevorsteht. Sie schnaubt leise. „Nein, noch 5 Minuten“, denkt sie. Ihre Hand tastet suchend in Richtung des penetranten Geräuschs. Ihre Finger strecken sich, schieben sich über die kühle Oberfläche des Plastikgehäuses ihres Funkweckers und suchen nach der schlafbringenden Erlösung. „5 Minuten... nur noch...“, sind die einzig klaren Gedanken, die sie fassen kann. Der Wecker gibt nach, das Geräusch verstummt. „...5 Minuten“, denkt sie und seufzt zufrieden.

Widerstrebend öffnen sich ihre Lider erneut. Stumm starrt sie durch das schattige, trübe Zwielicht zur Decke. Sie gähnt. Sekunden verstreichen, ehe sie einen Blick auf die digitale Anzeige neben ihr riskiert und sich selbst murren hört: „Nun gut. Dann eben 20 Minuten. Passt.“, denkt sie sich. Sie streckt sich genüsslich. Dann bringen ihre Ellenbogen ihren protestierenden Oberkörper langsam in eine aufrechtere Lage. Doch ihr Kopf sackt in den Nacken. *Nein*, denkt sie gequält. *Nein. Keine Lust. Ich habe einfach... keine Lust*. Sie seufzt und verharrt einen Moment in dieser Haltung. *Muss wohl. Bringt ja nichts,* ächzt sie leise vor sich hin.

Träge hieven unwillige Arme und Beine ihren bleiernen Körper über die Bettkante. Eine Hand greift widerstrebend nach achtlos über die Stuhllehne geworfenen Kleidungsstücken, die andere zerrt lustlos am Zugseil des Rollladens. Unter größtem Protest bahnen ihre Füße sich schließlich einen Weg über den Boden, treten dabei nachlässig verstreute Gegenstände und Papiere über den Teppich und tragen sie Richtung Bad.

„Was liegt an, Tina?“, fragt sie brummend ihr Gesicht in der fleckigen Spiegelfläche. „Nein, sag's nicht. Irgendwas mit Uni.“ Sie wendet sich von ihrem Spiegelbild ab.

*Keine Lust*, denkt sie, als ihre Hände die Hose über ihre Beine streifen. *Keine Lust*, während sie die Falten im Shirt glatt streicht. Ihr Blick begegnet ihr im Spiegel. Sie versucht, ihre Gedanken zu sammeln, sich zu konzentrieren auf... *Keine Lust.*

Seufzend kehrt sie zurück und verharrt im Rahmen der Zimmertür. Skeptisch wandert ihr Blick über Berge von Gegenständen, die sicherlich ihren angestammten Platz hätten, wenn sie nur nach einem für sie suchen würde… Eilig verstaute und aus Ordnern gefallene Aufzeichnungen lugen hinter Schränken und Kommoden hervor. Stifte liegen auf, hinter und unter dem Schreibtisch, umringt von haufenförmigen Ansammlungen aus unsortierten Klamotten. Ihr Blick fällt auf mehrere verstreut liegende Papiere. Eines wurde vom Wind aufgewirbelt und auf den kleinen Zierkaktus gespießt, der vertrocknet in krustiger Blumenerde auf dem Beistelltisch steht. Den vielen Formeln nach zu urteilen könnte es Statistik sein… trockener geht es kaum. Innerlich sackt sie ein wenig zusammen.

Dann gibt sie sich einen Ruck. Sie rafft sich auf, die Papiere vom Boden zu sammeln und versucht, sie zu ordnen. Media Richness, Reduced Social Cues, SIDE – die Aufzeichnungen sind mit Begriffen gefüllt, die ihr vage bekannt vorkommen, aber nie wirklich in ihrem Kopf abgespeichert wurden. Irgendwas mit Medien halt. Sie hat Mühe, die einzelnen Seiten in eine stimmige Reihenfolge zu bringen. Mehrfach muss sie sie hin- und herwenden, nebeneinander legen und angestrengt die Zeilen an ihrem Anfang und Ende vergleichen, bis sie schließlich aufgibt und sie nach einem müden Blick auf die Uhr eher willkürlich in ihren Ordner heftet.

Auf dem Weg zum Gruppentreffen grübelt sie lustlos über ihre letzte Sitzung nach. Über das Referat zur Media Equation, das sie vorbereiten müssen, und den unerträglich straffen und kleinschrittigen Zeitplan, den ein Kommilitone vorgeschlagen und für sie alle vorbereitet hat. „Gute Idee, findet ihr nicht“, fragte er stolz in die Runde. Sie wölbte die Brauen, verzichtete aber auf eine Antwort. Wie kann man so unentspannt sein? Es gibt Wichtigeres als Bücher, Skripte und ständige Lerntreffen. Wissen diese Leute überhaupt, wie man lebt?

Vor einer Haustür in der Sanderau bleibt sie stehen und hebt den Finger zum Klingelknopf. Prompt wird die Tür aufgerissen, und Max, der Kommilitone mit dem straffen Zeitplan funkelt sie herausfordernd an:

„Du kommst zu spät“, schnauzt Max. „Wir warten schon seit einer halben Stunde auf dich, du Faulpelz! Hättest dich wenigstens melden können.“

Sie hebt beschwichtigend die Hände. „Hey, hey! Sorry. Bin ja jetzt hier.“

Mit dem Kopf deutet er ruckartig in die Küche hinter sich. Sie folgt ihm zur Gruppe, die schweigend auseinanderrückt, um ihr Platz zu machen. Jemand sieht sie prüfend an:

„Verpennt?“

Sie schnauft abfällig. „Kommt vor.“

„Hast du deinen Teil vorbereitet?“, fragt Max.

Sie denkt nach. Nur schwach entsinnt sie sich der Inhalte, die sie gestern noch vor dem Zubettgehen überflogen hatte. Media Equation bedeutet, dass Menschen dazu neigen, Computer wie reale Personen zu behandeln. *Ziemlich selbsterklärend*, beschloss sie, und verzichtete deshalb darauf, sich irgendwelche Notizen zu machen.

„Ein wenig“, erwidert sie ausweichend, ohne zu überlegen. „Das Wichtigste eben.“ Insgeheim hofft sie, dass ihr weitere Nachfragen erspart bleiben.

Das Treffen verläuft so, wie sie es erwartet hatte: Sinnloses Beisammensitzen mehr oder weniger motivierter Menschen, die sich über Aufteilung, Reihenfolge und Inhalt der Referatsteile zanken. Sie hält sich zurück, besonders viel weiß sie ohnehin nicht. Während die anderen miteinander reden, blättert sie lustlos durch ihre unsortierten Unterlagen. *Kreative Ordnung in deinem Ordner*, denkt sie selbstironisch. Dann erzählt jemand, auf welch einfallsreiche Weise er seinen Teil vortragen wolle und verweist dabei auf eine Textstelle, die sie gar nicht finden kann. *Hm, blöd. Wohl daheim vergessen.* *Egal, bis morgen wird sich bestimmt alles finden*. *Habe mich allerdings schon mehrmals bei so etwas verschätzt...* Ob sie ihren Teil versuchsweise vortragen könne, fragt plötzlich der straff terminierte Kollege Max. „Nein“, erwidert sie und verdreht innerlich die Augen. „Aber ich werde ihn bis zur Präsentation morgen fertig haben.“ *Das heißt dann, wenn er wirklich wichtig ist*, fügt sie stumm hinzu.

Die Gruppe beschließt eine endgültige Reihenfolge und diskutiert ein letztes Mal die konkreten Inhalte. Ob sie mit dem Ergebnis zufrieden sei, wird sie gefragt. Sie hebt die Schultern, nickt dann. Eigentlich interessiert sie das Ganze nicht sonderlich. Wird schon schiefgehen.

Wieder zu Hause, schleudert sie den Rucksack zu Boden und holt ihr Handy hervor. Sie liest die Nachricht eines Freundes, der sie fragt, ob sie Lust hätte, mit ihm einen Film zu schauen. Sie zögert. Eigentlich sollte sie an ihrer Präsentation arbeiten. Sie besteht das Seminar nur, wenn sie eine halbwegs passable Präsentation abliefert. Ohne Referat kein bestandenes Seminar, also auch kein Notenbonus. Aber den Notenbonus könnte sie wirklich gut gebrauchen. Unschlüssig lässt sie das Handy sinken und blickt auf den Rucksack mit ihren Referatsmaterialien. *Erst mal etwas essen*, denkt sie. Danach kann sie immer noch entscheiden, was sie macht.

Während der Mahlzeit vibriert ihr Handy. Eine Nachricht vom eifrigen Kommilitonen Max: Er erinnert die Gruppe unter Einsatz einiger überflüssiger Satzzeichen daran, dass sie für morgen unbedingt und unter allen Umständen „an alles Wichtige denken müssen, das ist wirklich, wirklich wichtig, sonst stehen wir echt doof da!!!!!“ Sie grinst. *Fünf Ausrufungszeichen. Rufzeichen sind keine Rudeltiere… Wissen diese Leute, wie man lebt?*

Sie stapelt das Geschirr übereinander und schiebt es beiseite. In ihrem Zimmer lässt sie sich aufs Bett sinken. Zum Rhythmus ihrer trommelnden Finger erwägt sie, ob sie zu ihrem Freund gehen und den Film schauen oder lieber den Referatsteil vorbereiten sollte. Mehrere Augenblicke lang starrt sie in die Leere vor ihr. Die Entscheidung fällt ihr schwer. *Ach*, denkt sie sich, d*er Tag ist noch lang*, und springt auf. Sie nimmt die Jacke vom Haken. *Und für die Arbeit brauche ich Motivation, positive Energie*, denkt sie sich beschwingt. *Die kommt nicht von uninspiriertem Rumhängen über blöden Büchern!* Hinter ihr fällt die Tür ins Schloss, als sie sich gutgelaunt auf den Weg zu ihrem Freund macht.

Früh am Morgen kehrt sie mit dröhnendem Schädel zur Wohnung zurück. Vorsichtig öffnet sie die Haustür, schleicht zu ihrem Zimmer und schlüpft aus den Schuhen. Ihre Mitbewohnerin reiht ihre stets akkurat entlang der Wand auf, sie hingegen verteilt ihre in überschaubarem Durcheinander vor ihrer Zimmertür. Mit bloßen Zehen tastet sie sich über den kaum sichtbaren Teppich zu ihrem Bett. Dort zieht sie sich aus, lässt ihren Blick kurz durch den völlig überfüllten Raum gleiten und verstaut ihre Klamotten schließlich achselzuckend als Knäuel in einem schmalen Spalt zwischen der Schrank- und Zimmerwand. *Hat Platz und Halt*, denkt sie.

Erschöpft sinkt sie aufs Laken, presst eine Hand gegen die Schläfe und schließt die Augen. *Was für ein geiler Abend! Besser hätte er nicht laufen können. Vielleicht wiederhole ich das morgen, direkt nach...* Plötzlich reißt sie die Augen auf. Das Referat! Ihr Vortrag!

Sie schnellt hoch. Der Rucksack, wo ist er? Sie knipst das Licht an, starrt panisch auf das gewohnte Chaos, versucht, sich mit Blicken allein zu orientieren. Keine Chance! Hastig wühlt sie sich durch Berge getragener Hosen, Shirts, diverser Papiere und sonstigen Zeugs, das sich irgendwie, irgendwo in das vertraute Durcheinander fügt. *Verdammt. Verdammt! Warum liegt denn alles durcheinander? Idiot! Du wolltest gestern schon aufräumen!* Wieder und wieder hält sie inne, versucht Ordnung zu schaffen, schaufelt dabei jedoch bloß Gegenstände von einem Haufen auf den nächsten. Sie schluckt schwer. *Das ist doch nicht möglich!* *Wo habe ich das Ding hingestellt?* Sie dreht sich ratlos im Kreis, fühlt sich aufgeschmissen, irgendwie überrumpelt. Plötzlich ertastet eine Hand unter der achtlos hingeworfenen Jacke die Tragelasche des Rucksacks. Sie schließt die Augen, stößt erleichtert die Luft aus. Endlich! *Verfluchtes Referat!* Sie holt die mittlerweile stark geknitterten Seiten hervor, kneift die Augen zusammen und überfliegt mit trübem Blick eilig die Absätze. Konzentriert versucht sie, sich Inhalte zu merken, kaut nachdenklich auf ihrer Lippe und starrt gelegentlich an die Wand, darum bemüht, sich Fetzen des Geschriebenen einzuprägen. *Mann,* z*iemlich viel*. *Was für ein Stress*, grübelt sie müde.

Sie hält inne.

Und lässt die Papiere sinken.

*Aber schon alles irgendwie selbsterklärend, oder?,* denkt sie. *Wozu brauche ich Notizen? Wie schwer kann das schon sein? Bin ich eben spontan und denke mir den Text aus, wenn es soweit ist,* überlegt sie. *Stand-Up-Science. Kommt eh besser als diese auswendig gelernte Leierei.* *Passt. Wird schon schiefgehen,* spornt sie sich selbst an.

# Adjectives used for the SCIS - competence and SCIS - warmth in Study 1

German original [and English translation].

competence: kompetent, intelligent, fleißig, entschlossen [competent, intelligent, dilignet, determined]

warmth: sympathisch, hilfsbereit, aufrichtig, warm, freundlich [likeable, helpful, sincere, warm, kind]

control: sportlich, Sinn für Humor, musikalisch [athletic, sense of humor, musical]

# Adjectives used for the SCIS - conscientiousness in Study 2

German original [and English translation].

ausdauernd, willensstark, diszipliniert, organisiert, zuverlässig, ziellos, faul, chaotisch, unordentlich, unpünktlich [persistent, strong-willed, disciplined, organized, reliable, aimless, lazy, chaotic, untidy, unpunctual]
